# Feasibility of an Indigenous Food Is Medicine Program for Patients With Heart Failure in Rural Navajo Nation: The MUTTON-HF Nonrandomized Clinical Trial

**DOI:** 10.1001/jamanetworkopen.2025.56117

**Published:** 2026-02-06

**Authors:** Lauren A. Eberly, Carmen George, Sharon Sandman, Denee Bex, Matt Chandra, Kaitlyn Shultz, Ada Tennison, Rebecca Wickre, Bennett Wickre, Larissa Morgan, Leah Gray, Mackenzie Bolas, Benjamin Feliciano, DezBaa Damon-Mallette, Erica Lindsey, Jacob Manche, Pamela Detsoi-Smiley, Paula Mora, Maricruz Merino, Sonya S. Shin

**Affiliations:** 1 Gallup Indian Medical Center, Indian Health Service, Gallup, New Mexico; 2 Division of Cardiovascular Medicine, Department of Medicine, Hospital of the University of Pennsylvania, Philadelphia; 3 Penn Cardiovascular Outcomes, Quality, and Evaluative Research Center, Cardiovascular Institute, University of Pennsylvania, Philadelphia; 4 Penn Cardiovascular Center for Health Equity and Social Justice, University of Pennsylvania, Philadelphia; 5 Leonard Davis Institute of Health Economics, University of Pennsylvania, Philadelphia; 6 Division of Global Health Equity, Brigham and Women’s Hospital, Boston, Massachusetts; 7 Tumbleweed Nutrition LLC, Farmington, New Mexico; 8 Tocabe Inc, Denver, Colorado; 9 Division of Innovations and Improvement, Office of Quality, Indian Health Service Headquarters, Rockville, Maryland; 10 Department of Global Health and Social Medicine, Harvard Medical School, Boston, Massachusetts

## Abstract

**Importance:**

The ongoing consequences of settler colonialism produce adverse structural drivers, particularly nutrition insecurity, that contribute to cardiovascular health disparities among Indigenous populations. There is increased focus in Native communities to reclaim traditional precontact foods to improve health. Therefore, a locally sourced, Indigenous, medically tailored meal delivery program—MUTTON-HF (Medically Utilized Tailored Traditional Foods to Optimize Nutrition in Heart Failure)—was developed to improve health outcomes.

**Objective:**

To determine implementation outcomes, including feasibility and acceptability, as well as to explore preintervention vs postintervention health measures of a medically tailored meal program incorporating traditional foods and recipes for patients with heart failure in rural Navajo Nation.

**Design, Setting, and Participants:**

The single-arm pilot nonrandomized clinical trial was conducted from October 7, 2024, to February 3, 2025, to evaluate implementation and health outcomes of the MUTTON-HF program. Participants included adults (≥18 years) with a diagnosis of heart failure who were receiving care at one of 2 Indian Health Service sites in rural Navajo Nation.

**Intervention:**

Patients received 14 culturally and medically tailored meals weekly (2 meals daily) for 4 weeks.

**Main Outcomes and Measures:**

The primary outcomes were intervention feasibility and acceptability, assessed with surveys, qualitative interviews, and programmatic data at 30 days. Intervention feasibility was determined by evaluating the number and percentage of meal boxes successfully received by each patient. Acceptability was assessed using the Acceptability of Intervention Measure (AIM) (score range, 4-20), patient program ratings (range, 1-10), and the Net Promoter Score. Secondary outcomes, which were assessed via surveys and medical record review, included intervention adoption and fidelity, feasibility for community partners (including farmers and ranchers, using the Feasibility of Intervention Measure [score range, 4-20]), and preintervention vs postintervention health measures (eg, clinical biomarkers, food insecurity [based on the US Department of Agriculture 6-item Short-Form Food Security Survey Module], 12-item Kansas City Cardiomyopathy Questionnaire [KCCQ] scores, and Cultural Connectedness Scale [CCS] scores).

**Results:**

This study enrolled 20 American Indian patients (mean [SD] age, 58.2 [11.7] years; 13 were male [65.0%]) residing in communities exceeding a 136-km radius in Arizona and New Mexico. Patients had a mean (SD) left ventricular ejection fraction of 40.0% (16.0%). Of the 80 weekly meal boxes, 72 (90.0%) were successfully received by patients. The mean (SD) AIM score was 16.9 (3.1), the mean (SD) patient program rating was 8.6 (1.6), and the Net Promoter Score was 45.0%. Most patients (17 [85.0%]) reported they were likely to change their diet to be healthier moving forward. Mean (SD) Feasibility of Intervention Measure scores were 19.8 (0.5) for community farmers and ranchers and 20 (0) for community partners. Significant preintervention to postintervention improvements were observed for food security (the number of patients who were food secure increased from 8 [40.0%] to 17 [85.0%]), KCCQ physical limitation (mean [SD], from 59.6 [31.3] to 82.7 [21.9]) and social limitation (mean [SD], from 74.6 [24.1] to 83.8 [25.0]) scores, CCS Traditions subscore (mean [SD], from 7.2 [2.9] to 7.9 [3.0]), and weight change among patients with obesity (mean [SD], −2.3 [3.3] kg).

**Conclusions and Relevance:**

In this nonrandomized clinical trial, the MUTTON-HF intervention incorporating Indigenous recipes and locally sourced Native food was feasible and acceptable for patients with heart failure in rural Navajo Nation. These findings will inform a future randomized clinical trial to evaluate the effectiveness of this intervention to advance Indigenous cardiovascular health and food sovereignty.

**Trial Registration:**

ClinicalTrials.gov Identifier: NCT06675331

## Key Points

**Question:**

Among Navajo patients with heart failure living rurally on the reservation, is a medically tailored meal delivery program incorporating Indigenous foods and recipes feasible and acceptable?

**Findings:**

This nonrandomized clinical trial included 20 American Indian patients with heart failure receiving care at 2 Indian Health Service sites in rural Navajo Nation. A community-designed, Indigenous, medically tailored meal program was implemented; the intervention was deemed both feasible (90% of weekly meal boxes received by patients) and acceptable (mean Acceptability of Intervention Measure score, 17 of 20).

**Meaning:**

A Native and locally sourced meal delivery program of medically and culturally tailored meals was found to be a feasible and acceptable intervention for Navajo patients with heart failure who reside rurally.

## Teaser

This nonrandomized clinical trial assesses the feasibility and acceptability of a medically tailored meal delivery program incorporating traditional foods and recipes for Navajo patients with heart failure.

## Introduction

Indigenous populations in the US experience substantial cardiovascular health inequities.^1,2^ Cardiovascular disparities are rooted in colonial legacies of resource extraction, land theft, structural violence, and exclusionary policies.^3,4^ The enduring consequences of settler colonialism have produced adverse structural drivers—in particular, nutrition insecurity—that contribute to poor cardiovascular health among Indigenous populations.^4,5,6,7,8,9^ Improving the health of Indigenous people requires addressing upstream structural drivers of health, including nutrition insecurity.

Dietary factors are an important cause of hospitalizations in patients with heart failure (HF).^10^ Food Is Medicine (FIM) programs, such as direct dietary support through produce prescriptions or medically tailored meals (MTMs), may improve cardiovascular outcomes and disease-specific quality of life.^11,12^ As Native communities gain momentum in reclaiming Indigenous foods, community-designed programs that improve access to healthy foods, reclaim precolonial foods, and support local food systems could advance health while also empowering tribal communities and promoting food sovereignty. However, more evidence on FIM programs in Native communities is needed.^13^ Additionally, operationalizing FIM programs across tribal reservations can pose logistical challenges due to rurality, vast distances between patients, lack of paved roads, and environmental infrastructure.^14^

Using community-based participatory methods, we designed a Native-sourced, culturally and medically tailored meal (CMTM) program (Medically Utilized Tailored Traditional Foods to Optimize Nutrition in Heart Failure [MUTTON-HF]) to improve outcomes for patients with HF in rural Navajo Nation.^15^ Before undertaking a randomized clinical trial, we performed a feasibility study to optimize the intervention. The aim of this pilot trial was to evaluate implementation outcomes including feasibility, acceptability, adoption, and fidelity of MUTTON-HF among patients with HF at 2 Indian Health Service (IHS) sites in rural Navajo Nation. We hypothesized that MUTTON-HF would be feasible and acceptable for Diné (Navajo) patients and implementing partners.

## Methods

This nonrandomized clinical trial was approved by the Navajo Nation Human Research Review Board (NNR 24.525) and by the Mass General Brigham Institutional Review Board. Written informed consent was obtained from all patients. The full trial protocol is available on the MUTTON-HF trial website,^15^ in Supplement 1, and at ClinicalTrials.gov (NCT06675331). This study followed the Transparent Reporting of Evaluations With Nonrandomized Designs (TREND) reporting guideline as well as the Consolidated Criteria for Reporting Qualitative Research (COREQ) reporting guideline.

As part of an IHS Office of Quality Innovations Award, this study used community-based participatory research (CBPR) methods to improve the quality of HF care.^16^ CBPR informed engagement to identify the community priority (ie, addressing food insecurity), codesign the intervention (ie, locally sourced, traditional Diné foods and recipes), select study outcomes (ie, culturally relevant tools), and equitably value community partners and participants. Additionally, we shared results and conducted listening sessions with pilot participants, community partners, and practitioners, which informed this article and a dissemination plan for the future randomized clinical trial.^17^ The sample size and intervention duration were determined based on similar pilot MTM studies in other settings and deemed sufficient by stakeholders to assess outcomes given budgetary constraints.^18^

### Study Setting and Sites

This study was conducted from October 7, 2024, to February 3, 2025, at 2 IHS ambulatory clinic sites in Navajo Nation: the largest IHS site serving the Navajo Nation and a smaller, rural site.^19,20^ These sites have a combined catchment area over a 136-km radius (to convert kilometers to miles, divide by 1.6).^19,20^

FIM is a long-standing belief in Diné culture, where traditional foods are often used and served in ceremonies. Food is considered sacred. Many traditional foods consumed as part of routine diets are central to healing, and the cultivation of food as living plants and animals is considered a sacred task on the part of ranchers and growers.

### Study Population

The study population comprised individuals with a diagnosis of HF with reduced ejection fraction (based on *International Classification of Diseases, Tenth Revision* codes) and a hospitalization in the last 12 months who were actively engaged in care at one of the 2 IHS facilities. Active engagement was defined as having a primary care physician at one of the sites, as well as a clinic visit and a prescription in the last 12 months. Patients living in an acute rehabilitation or skilled nursing facility or receiving hospice care were excluded. We used purposeful sampling based on community^21^ and age to ensure broad representation.

### Design of the Mutton-HF Program

The design of the MUTTON-HF program is illustrated in eFigure 1 in Supplement 2. CMTMs were designed by a Diné registered dietitian nutritionist (D.B.)^22^ to incorporate traditional Diné foods and recipes. Meals met American Heart Association Dietary Approaches to Stop Hypertension recommendations.^23,24^ In an effort to advance food sovereignty and increase acceptability, we sourced meat and produce from Diné farmers and ranchers.^25^ Tocabe, a Native-owned food organization, produced, packaged, and shipped the meals to the Gallup Community Pantry, the centralized food hub.^26,27^

### Meal Distribution, Storage, and Preparation

Because preliminary data showed that more than 95% of patients with HF at the 2 IHS sites use post office boxes rather than a physical mailing address, we developed a delivery and distribution network with community partners. Patients completed an intake form that assessed delivery preference, persons authorized for pickup, and home resources (eg, electricity, freezer, and microwave). As a default, patients or proxies picked up meals at the pantry on a weekly basis. For more remote patients, meals were delivered to minihubs (eg, Chapter Houses) for pickup.^21^ For those with neither transportation nor a support person, meals were delivered by a Community Health Representative (CHR) or a peer (another participant living nearby). Insulated boxes facilitated delivery and patient transport, limiting the need for coolers, ice, or dry ice. Meals included instructions for both microwave or stove heating (including propane-supported or camping stoves) to allow for patient preferences and resources.

### Intervention

Patients received 14 meals weekly (2 meals daily) for 4 weeks, followed by a cooking class to learn how to make CMTM recipes to help sustain healthy behaviors. As needed, appliances (eg, microwave or small freezer) were provided at no cost.

### Outcomes

Outcomes were assessed at the end of the intervention (ie, at 30 days). Surveys, medical record review, and qualitative interviews were used for assessment.

#### Primary Outcomes

The primary outcomes were intervention feasibility and acceptability. Both outcomes were assessed using quantitative and qualitative methods.

##### Feasibility

We assessed intervention feasibility by evaluating the number and percentage of meals delivered successfully to the Gallup Food Pantry, the number and percentage of meals successfully received by patients, and rates of successful delivery by delivery mechanism. Patients were also surveyed using a 5-item Likert scale to rate difficulty of meal delivery or pickup. We determined that a rate of successful delivery of greater than 75% would indicate program feasibility.

##### Acceptability

We evaluated intervention acceptability using the validated Acceptability of Intervention Measure (AIM) (score range, 4-20; scores >16 represent high acceptability) and patient rating of the program (rating range, 1-10; response to the question, “How likely is it from 1-10 that you would recommend this program to a community member?”), which was also used to calculate the Net Promoter Score.^28^ Semistructured patient interviews, guided by the Consolidated Framework for Implementation Research (CFIR), explored multiple constructs within each CFIR domain hypothesized by the study team and based on existing literature to be relevant to program acceptability and feasibility.^29^

#### Secondary Outcomes

##### Fidelity

To assess intervention fidelity, we evaluated the amount and percentage of meals consumed and shared. Fidelity was defined as at least 70% participants indicating that they ate most or all of the meals.

##### Adoption

To evaluate adoption, we used a postintervention 5-item Likert scale to survey participants regarding the likelihood that the program will change their diet to be healthier moving forward. We also asked participants about the likelihood of them adding any meals and recipes from the program to their diet in the future.

##### Feasibility and Acceptability of Supporting Local Food Systems

To assess intervention feasibility and acceptability of supporting local food systems, we used the validated Feasibility of Intervention Measure among food pantry, farming, and ranching partners.^28^ This measure provides a score ranging from 4 to 20, with values greater than 16 representing a highly feasible intervention.^28^ We also assessed the percentage of meals that included Native and Navajo ingredients.

##### Clinical Outcomes and Feasibility of Outcome Assessment

We piloted outcome assessments for the future randomized clinical trial, including laboratory biomarkers (comprehensive metabolic panel, lipid panel, hemoglobin A_1c_, prealbumin, B-type natriuretic peptide [BNP] or N-terminal pro-BNP), blood pressure, weight, body mass index (BMI), 12-item Kansas City Cardiomyopathy Questionnaire (KCCQ) score and its components,^30^ food security (US Department of Agriculture [USDA] 6-item Short-Form Food Security Survey Module [FSSM]),^31^ and cultural connectedness (using the Cultural Connectedness Scale [CCS])^32^ at baseline and after the intervention (ie, at 30 days). The postintervention USDA FSSM questionnaire was adapted to evaluate food security within the last 1 month (rather than 12 months). We assessed baseline diet quality using the 10-item Dietary Screener Questionnaire (DSQ) plus 1 additional question on traditional Diné foods.^33^ The CCS measures cultural connectedness in Indigenous populations and correlates with physical and mental health.^34^ For trial feasibility, we considered 80% or greater completion of preintervention and postintervention surveys and laboratory measures a success.

Additional exploratory outcomes included patient-reported general health status. All clinical outcomes and subgroup analyses were exploratory. Baseline and postintervention surveys are included in eAppendices 1 to 2 in Supplement 2, respectively. Interview guides for patients and for farmers and ranchers are included eAppendices 3 to 4 in Supplement 2, respectively.

### Statistical Analysis

We determined descriptive statistics of baseline characteristics, and we assessed differences from baseline to week 4 changes in continuous outcomes using the Wilcoxon signed rank test and proportions using the McNemar test or the Bowker symmetry test. We also performed prespecified subgroup analysis to evaluate changes in weight among those with baseline obesity (BMI ≥30 [calculated as weight in kilograms divided by height in meters squared]).

Semistructured interviews were audiorecorded, and transcripts were analyzed using an integrated approach to identify themes and patterns.^35^ Interviewers developed the initial codebook together, independently dual coded subsets of the transcripts to identify common themes, and then iteratively developed a final codebook, with periodic assessment of interrater reliability.

Two-sided *P* < .05 was considered statistically significant. SAS, version 9.4 (SAS Institute), was used for data analyses.

## Results

Of the 24 patients assessed for study eligibility, 20 were enrolled. Their mean age (SD) was 58.2 (11.7) years; 7 patients (35.0%) were female, 13 (65.0%) were male, and all 20 (100%) were American Indian. Baseline characteristics are summarized in Table 1, with baseline clinical data presented in Table 2^36^ and the eTable in Supplement 2.

**Table 1.** Baseline Characteristics of the Study Cohort

Characteristic	No. (%) (N = 20)^a^
**Demographic**	
Age, mean (SD), y	58.2 (11.7)
Sex	
Female	7 (35.0)
Male	13 (65.0)
State of residence	
New Mexico	16 (80.0)
Arizona	4 (20.0)
Race and ethnicity	
American Indian	20 (100)
Hispanic	1 (5.0)
Tribal affiliation	
Navajo	19 (95.0)
Zuni Pueblo	1 (5.0)
Cultural Connectedness Scale score, mean (SD)	29.3 (7.2)
Food security status (based on USDA FSSM score)	
High	8 (40.0)
Low	8 (40.0)
Very low	4 (20.0)
Language preferences	
Speak English well	18 (90.0)
Speak Navajo well	12 (60.0)
Read English well	20 (100)
Read Navajo well	3 (15.0)
Self-reported health status	
Poor	1 (5.0)
Fair	6 (30.0)
Good	11 (55.0)
Excellent	2 (10.0)
Exercise, mean (SD), min/wk	78.9 (85.2)
**Diet quality**	
Responsible for cooking	
No	2 (10.0)
Some	11 (55.0)
Yes	7 (35.0)
Servings of fruits and vegetables (based on the DSQ-10), mean (SD), cups/d^b^	
Fruits	1.1 (0.6)
Vegetables including fried potatoes	1.7 (0.4)
Vegetables not including fried potatoes	1.6 (0.4)
Fruits and vegetables including fried potatoes	2.8 (0.8)
Fruits and vegetables not including fried potatoes	2.7 (0.8)
Eat traditional Diné (Navajo) meals	
1 Time/d	3 (15.0)
3-4 Times/wk	2 (10.0)
2 Times/wk	1 (5.0)
1 Time/wk	1 (5.0)
2-3 Times/prior mo	2 (10.0)
1 Time/prior mo	9 (45.0)
Never	1 (5.0)
**Socioeconomic and household**	
How often do you do not have enough money for bills?	
Never	3 (15.0)
Rarely	8 (40.0)
Sometimes	7 (35.0)
Often	1 (5.0)
Missing	1 (5.0)
Working appliances	
Microwave	20 (100)
Stove	20 (100)
Freezer	18 (90.0)
Refrigerator	20 (100)
Reliable electricity	19 (95.0)
Running tap water	19 (95.0)
Water insecure^c^	4 (20.0)
Enrollment in food assistance programs in prior 12 mo	
SNAP	5 (25.0)
Commodities	1 (5.0)
WIC	1 (5.0)
Other	2 (10.0)
Participation in other community assistance programs in prior 12 mo	
Senior center	1 (5.0)
Meals on Wheels	1 (5.0)
Public health nurse	0
Community Health Representative	1 (5.0)
Local church	3 (15.0)
**Clinical**	
Weight, mean (SD), kg	103.7 (25.5)
BMI, mean (SD)	36.5 (10.4)
Blood pressure, mean (SD), mm Hg	
Systolic	122.7 (17.8)
Diastolic	76.4 (8.8)
Type of heart failure	
Ischemic	6 (30.0)
Nonischemic	14 (70.0)
Left ventricular ejection fraction, mean (SD), %	40.0 (16.0)
Comorbidities	
Hypertension	15 (75.0)
Atrial fibrillation	5 (25.0)
Coronary artery disease	9 (45.0)
Chronic kidney disease	7 (35.0)
Stage	
1	1 (5.0)
2	1 (5.0)
3	2 (10.0)
4	1 (5.0)
5 (End-stage kidney disease)	1 (5.0)
Receiving hemodialysis	1 (5.0)
Type 2 diabetes	11 (55.0)
Hyperlipidemia	6 (30.0)
Obesity	14 (70.0)
Prediabetes	7 (35.0)
Obstructive sleep apnea	6 (30.0)
KCCQ score, mean (SD)	
Summary total	69.6 (22.1)
Physical limitation	59.6 (31.3)
Symptom frequency	74.4 (25.1)
Quality of life	70.0 (24.5)
Social limitation	74.6 (24.1)

Abbreviations: BMI, body mass index (calculated as weight in kilograms divided by height in meters squared); DSQ-10, 10-item Dietary Screener Questionnaire; FSSM, 6-item Short-Form Food Security Survey Module; KCCQ, 12-item Kansas City Cardiomyopathy Questionnaire; SNAP, Supplemental Nutrition Assistance Program; USDA, US Department of Agriculture; WIC, Special Supplemental Nutrition Program for Women, Infants, and Children.

^a^ Unless indicated otherwise, values are presented as No. (%) of patients.

^b^ One patient had missing DSQ-10 data.

^c^ Validated water security indicators were used to determine water insecurity, with the following responses being consistent with water insecurity: answering “No” to the question “Do you have running (tap) water?”; answering “Disagree” for either statement “My tap water at home is safe to drink” or “My tap water at home is safe to cook with”; or answering “I never drink tap water” when asked “When you drink tap water, what is the main source of the tap water?” One patient had missing data on these questions.

**Table 2.** Changes in Clinical Biomarkers and Outcomes Before and After the Intervention

Measure	Value^a^		Change	Percent change	*P* value^b^
	Baseline	At 30 d			
Weight, kg	103.7 (25.5)	102.4 (25.3)	−1.3	−1.2	.17
Blood pressure, mm Hg					
Systolic	122.7 (17.9)	121.6 (12.3)	−1.1	−0.9	.69
Diastolic	76.4 (8.8)	77.4 (7.8)	0.9	1.2	>.99
NT-BNP, pg/mL^c^	1518.4 (3339.3)	2002.5 (5884.8)	484.1	31.9	.52
C-reactive protein, mg/dL	0.61 (0.5)	0.59 (0.6)	−0.02	−3.3	.90
Hemoglobin A_1c_, %	7.0 (2.1)	6.9 (1.9)	−0.1	−0.7	.64
Cholesterol, mg/dL					
Total	128.8 (36.7)	124.8 (31.4)	−4.0	−3.1	.60
LDL	64.0 (28.5)	62.9 (26.2)	−1.1	−1.7	.74
HDL	44.1 (13.7)	43.6 (9.2)	−0.5	−1.2	.76
Triglycerides, mg/dL	147.8 (77.7)	127.1 (54.4)	−20.7	−43.3	.45
Prealbumin, mg/dL	20.0 (4.3)	21.1 (4.2)	1.1	5.9	.06
Albumin, g/dL	4.01 (0.3)	4.03 (0.3)	0.02	0.5	.67
CCS score					
Total	29.3 (7.2)	31.2 (6.9)	1.9	6.3	.08
Traditions	7.2 (2.9)	7.9 (3.0)	0.7	9.8	.04
Spirituality	6.2 (1.8)	6.8 (1.5)	0.6	9.8	.07
Identity	16.0 (3.3)	16.3 (3.9)	0.3	1.9	.42
KCCQ score					
Summary	69.6 (22.1)	78.2 (22.0)	8.6	12.3	.06
Physical limitation	59.6 (31.3)	82.7 (21.9)	23.1	38.8	<.001
Symptom frequency	74.4 (25.1)	81.4 (28.5)	7.0	9.4	.05
Quality of life	70.0 (24.5)	65.0 (27.4)	−5.0	−7.1	.30
Social limitation	74.6 (24.1)	83.8 (25.0)	9.2	12.3	.045
USDA FSSM total score	2.2 (2.1)	0.7 (1.1)	−1.5	−69.8	.02
Food security category, No. (%) of patients					
Food secure	8 (40.0)	17 (85.0)	NA	NA	.02
Low food security	8 (40.0)	3 (15.0)			
Very low food security	4 (20.0)	0			

Abbreviations: BMI, body mass index; CCS, Cultural Connectedness Scale; eGFR, estimated glomerular filtration rate; FSSM, 6-item Short-Form Food Security Survey Module; HDL, high-density lipoprotein; KCCQ, 12-item Kansas City Cardiomyopathy Questionnaire; LDL, low-density lipoprotein; NA, not applicable; NT-BNP, N-terminal pro-B-type natriuretic peptide; USDA, US Department of Agriculture.

SI conversion factors: To convert NT-BNP to ng/L, multiply by 1; C-reactive protein and prealbumin to mg/L, multiply by 10; total, HDL, and LDL cholesterol to mmol/L, multiply by 0.0259; triglycerides to mmol/L, multiply by 0.0113; and albumin to g/L, multiply by 10.

^a^ Unless indicated otherwise, values are presented as the mean (SD).

^b^ *P* values derived from the paired *t* test vs the McNemar test as appropriate.

^c^ Two patients had BNP at baseline, with 1 patient having pro-BNP at 30 days. Given this, BNP values were converted to NT-BNP using the following equation from Ishihara et al^36^: log NT-proBNP = 1.21 + 1.03 × log BNP − 0.009 × BMI − 0.007 × eGFR.

Twelve patients (60.0%) experienced food insecurity, and 9 (45.0%) participated in a food assistance program (eg, the Supplemental Nutrition Assistance Program or the Special Supplemental Nutrition Program for Women, Infants, and Children). Most patients (19 [95.0%]) had electricity and running tap water, but 4 (20.0%) were water insecure.^37^ Two patients (10.0%) did not have a freezer and were provided one. No patients were lost to follow-up or had missing outcome data. Patients resided in various communities spanning 2 states (New Mexico or Arizona), with a greater than 136-km radius (ie, a distance of 137.6 km North to South and 91.2 km West to East) (Figure).

**Figure.**
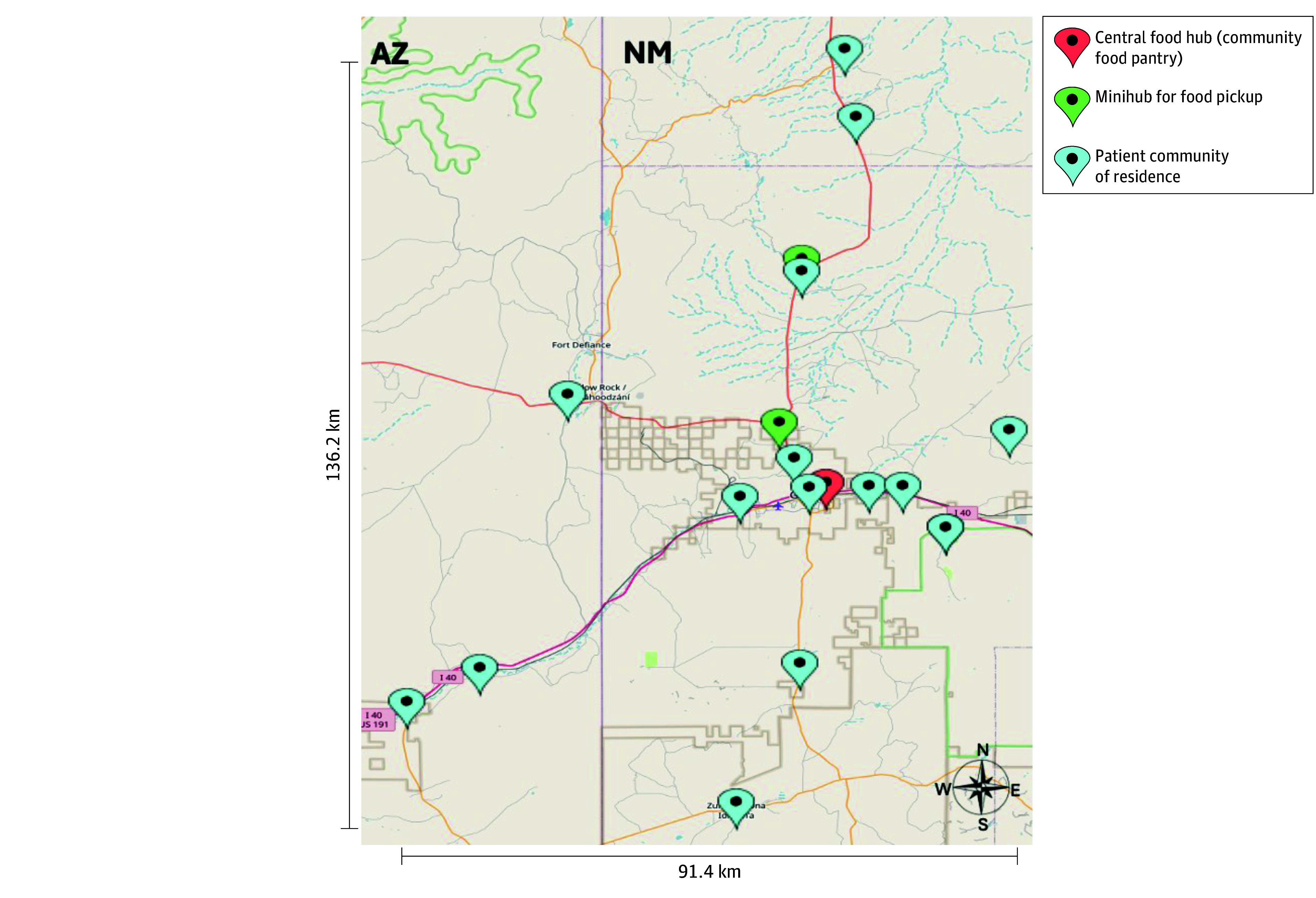
Map of Patient Communities of Residence and Food Hubs in Rural Eastern Navajo Nation for the MUTTON-HF Pilot Trial To convert kilometers to miles, divide by 1.6.

Patients had a mean (SD) left ventricular ejection fraction of 40.0% (16.0%), with mean (SD) BMI at baseline of 36.5 (10.4). Common comorbidities included hypertension (n = 15 [75.0%]), obesity (n = 14 [70.0%]), and type 2 diabetes or prediabetes (n = 18 [90.0%]). The mean (SD) KCCQ summary score was 69.6 (22.1).

### Implementation Outcomes

Primary and secondary implementation and clinical outcomes are described herein. Implementation outcomes are also summarized in Table 3 and eFigure 2 in Supplement 2.

**Table 3.** MUTTON-HF Patient-Related Implementation Outcomes

Outcome measure	Value (N = 20)^a^
**Feasibility**	
No./Total No. (%) of weekly meal boxes successfully received by patients	72/80 (90.0)
How easy would you rank getting the meals?	
Very difficult	0
Somewhat difficult	1 (5.0)
Neither easy nor difficult	1 (5.0)
Easy	5 (25.0)
Very easy	13 (65.0)
**Acceptability**	
AIM score (range, 4-20), mean (SD)	16.9 (3.1)
Patient program rating (range, 1-10), mean (SD)	8.6 (1.6)
Net Promoter Score, %	45.0
**Adoption**	
How likely is it that this program will change your diet to be healthier moving forward?	
Very unlikely	0
Somewhat unlikely	0
Neither likely nor unlikely	3 (15.0)
Somewhat likely	9 (45.0)
Very likely	8 (40.0)
How likely is it that you will add any of the meals or recipes from this program to your ongoing diet in the future?	
Very unlikely	1 (5.0)
Somewhat unlikely	2 (10.0)
Neither likely nor unlikely	4 (20.0)
Somewhat likely	5 (25.0)
Very likely	8 (40.0)
**Fidelity**	
In terms of sharing meals, how much of the meals were shared with others?	
None	11 (55.0)
Less than half	6 (30.0)
About half	1 (5.0)
Most	2 (10.0)
How much of the meals were consumed and not thrown out?	
None	1 (5.0)
Less than half	2 (10.0)
More than half	9 (45.0)
All	8 (40.0)

Abbreviation: AIM, Acceptability of Intervention Measure.

^a^ Unless indicated otherwise, values are presented as the No. (%) of participants.

#### Primary Outcome: Intervention Feasibility and Acceptability

Patients received 1 meal box per week, each containing 14 CMTMs (2 meals daily) for 4 weeks. All 80 meal boxes (100%) were successfully delivered to the central hub, and 72 of the 80 weekly meal boxes (90.0%) were received by the patients. Separated by delivery method, 50 of 58 meal boxes (86.2%) were picked up at the pantry (n = 15 patients). Other meal boxes reached patients via pickup at minihubs (8 of 8 boxes [100%]; n = 2 patients), peer delivery (4 of 4 boxes [100%]; n = 1 patient), and CHR delivery (10 of 10 boxes [100%]; n = 3 patients); 1 patient initially opted for pickup at the food pantry, but after 2 weeks requested CHR delivery of the remaining meals. Eighteen patients (90.0%) reported that it was very easy or somewhat easy to get the meals. The mean (SD) AIM score was 16.9 (3.1), the mean (SD) patient program rating was 8.6 (1.6), and the Net Promoter Score was 45.0%.

On qualitative interviews, MUTTON-HF was well received. Patients felt that getting the meals was easy, and they enjoyed the program. Several patients described having improved energy and functional status. One patient reported that “this program changed everything for me” after losing weight and gaining a better understanding of what he should be eating. Although a few patients noted a period of adjustment to the low-sodium diet, most described learning that healthy food could be tasty (one patient commented, “I didn’t know that healthy food could taste so good”). Many patients noted the program connected them to their culture, reminding them of their elders’ cooking, which brought tremendous comfort. The majority of patients expressed pride that the food was locally sourced. For many, the program alleviated financial stress, allowing them to catch up on forgone or delayed bills. Challenges included consumption during holidays, inclement weather, and (for 1 patient) meal adherence during a cultural ceremony.

#### Secondary Outcomes

##### Fidelity and Adoption

With regard to intervention fidelity and adoption, the majority of patients (17 [85.0%]) reported that they ate most or all of the meals. Seventeen patients (85.0%) reported that they were very likely or somewhat likely to change their diet to be healthier moving forward, and 13 (65.0%) were very likely or somewhat likely to adopt MUTTON-HF meals and/or recipes after the program.

##### Feasibility for Community Partners

We surveyed 9 community partners, including 2 pantry staff members, 4 farmers, 2 ranchers, and 1 Tocabe staff member. The mean (SD) Feasibility of Intervention Measure score (out of 20) was 20 (0) for the pantry staff members, 19.7 (0.6) for the farmers, 20 (0) for the ranchers, and 20 (0) for the Tocabe staff member. Of the 21 prepared meal types, all 21 (100%) were sourced from Native suppliers; 15 of 21 meals (71.4%) were from local Navajo farmers or ranchers specifically.

##### Clinical Outcomes

All 20 patients (100%) completed preintervention and postintervention surveys and laboratory measures. Changes in patient-reported and clinical outcomes are presented in Table 2. Very low food security decreased from 4 patients (20.0%) to 0, while food security increased from 8 patients (40.0%) to 17 (85.0%) (*P* = .006). The mean (SD) KCCQ summary score increased from 69.6 (22.1) at baseline (fair to good) to 78.2 (22.0) at 30 days (good to excellent) (*P* = .06), with significant increases in mean (SD) KCCQ physical limitation score from 59.6 (31.3) to 82.7 (21.9) (*P* < .001) and in mean (SD) KCCQ social limitation score from 74.6 (24.1) to 83.8 (25.0) (*P* = .045). Sixteen patients (80.0%) had stable or improved self-reported health status at 30 days from baseline. Mean (SD) CCS and subscores increased from baseline, with the greatest increase in the Traditions subscore from 7.2 (2.9) to 7.9 (3.0) (*P* = .04). Patients with baseline obesity (n = 14) experienced a mean (SD) change of −2.3 (3.3) kg (*P* = .03) and a mean (SD) weight loss of 2.1% (3.2%).

## Discussion

To our knowledge, this is the first study to evaluate implementation outcomes of a Native-sourced CMTM program incorporating traditional Indigenous foods for patients with heart failure. We demonstrated intervention feasibility and acceptability among Navajo patients living with HF and among community partners. Although our primary end points were feasibility and acceptability, we observed significant improvements in food security, KCCQ physical and social limitation scores, and weight among patients with obesity at baseline. These pilot results have informed the final design of our future randomized clinical trial, which will evaluate the effectiveness of an 8-week MUTTON-HF intervention on patient-reported and clinical outcomes compared with usual care.^17^

The intersection of settler colonialism and rurality creates challenges for reservation communities where vast distances, inadequate water infrastructure, unpaved roads, and environmental toxins pose barriers to food access. Meal delivery programs for cardiovascular health have been implemented and evaluated for other populations, predominately in urban settings.^11,38,39^ In our population, similar to other rural tribal communities, infrastructure issues, including lack of paved roads and physical addresses, immense distances, and limited transportation, introduce potential challenges. The MUTTON-HF program was designed with community partners to overcome such challenges and reach patients with varying needs spanning remote areas. Lessons learned include the importance of final-mile strategies, such as providing needed appliances and flexible delivery options. The findings of this study suggest that this model is feasible; we hope that these results will support such models to be similarly designed and adapted by other tribal communities to fit their needs, lower food insecurity, and advance Indigenous health.

Cardiovascular health inequities among Indigenous populations are rooted in racism, sociopolitical and environmental injustices, and structural violence. Before colonization, Indigenous peoples lived a healthy lifestyle with consumption of traditional healthy foods.^40^ However, similar to many Indigenous communities, pervasive nutrition insecurity in Navajo Nation is a direct result of forced government relocation, broken treaty obligations, exclusionary governmental policies, and community disinvestment.^4,5,6,7,41,42,43,44^ Food insecurity leads to high rates of obesity, dyslipidemia, and diabetes and is independently associated with poor cardiovascular health.^45^ Efforts to rectify historic and ongoing injustices are necessary to address structural drivers of health.^5,46,47^ FIM programs, such as MUTTON-HF, which address food insecurity as an integral part of health care services may combat the disproportionate burden of disease in Native communities by reclaiming traditional Indigenous ways of living, including improving access to precontact foods.^48,49,50,51,52,53,54,55,56^

Leveraging the protective assets of Indigenous communities is critical to advancing health equity.^5^ After a 4-week CMTM program incorporating traditional Diné meals, cultural connectedness increased in this study (as assessed with CCS scores). Cultural connectedness has been shown to be an important Indigenous determinant of health.^5,34,57,58,59,60^ Beyond providing medically tailored sodium-restricted meals, MUTTON-HF is designed to strengthen cultural connectedness, which improves health outcomes by increasing meal adherence and also enhancing emotional and spiritual well-being.^34^ Future research is needed to understand unique considerations of traditionally sourced, culturally grounded FIM programs, both in terms of program operationalization as well as mediating and moderating effects.

Community-clinic linkages to advance Indigenous health must center around active community participation and tribal sovereignty. The MUTTON-HF program was designed to support local farmers and ranchers to source meat and produce. Such programs may improve not only patient-level health but also community health through promoting food sovereignty and empowering tribal communities.

Although this pilot trial was not powered to evaluate clinical outcomes, we found significant improvement in components of the KCCQ score with a trend toward improved KCCQ summary scores. This finding is consistent with prior MTM studies demonstrating improvements in KCCQ scores in other populations.^11^ Our larger randomized clinical trial to evaluate the effectiveness of an 8-week MUTTON-HF intervention is underway.^17^ Results of the current pilot trial, including feedback on each meal and listening sessions with pilot participants and community members, have informed trial design and intervention optimization for the larger trial.^17^

### Limitations

This study has some limitations, including its small sample size, short intervention duration, and setting. The results may not be generalizable. We relied on a food pantry with considerable freezer space, which may not be available in all settings. Expansion to other settings would require adaption to fit the local context, cuisine, and cultural preferences. Future research is warranted to understand how factors such as family dynamics and cultural preferences may influence program implementation, patient participation, and treatment response. Although some patients lacked running tap water or a refrigerator and/or freezer, most had stable electricity. Ongoing evaluation as the program expands to patients without electricity is necessary to understand scalability and generalizability. FIM programs should also consider syndemic water and food insecurity in future research. Further implementation research is needed to demonstrate sustainability and inform widespread adoption. Effectiveness and implementation data from our future comparativeness effectiveness trial will inform expansion and sustainable adoption of similar programs throughout the IHS nationally.

## Conclusions

In this nonrandomized clinical trial of a community-designed Indigenous FIM program, 4 weeks of CMTM delivery was feasible and acceptable for patients with HF in rural Navajo Nation. Centering communities and protective assets of Indigenous communities is critical to advance health equity. Promising outcomes of this study, including weight loss among those with obesity and improvements in HF symptoms, highlight the need for a randomized clinical trial to rigorously evaluate effectiveness and implementation requirements for sustainability.

## Notes

Supplement 1.
Trial Protocol

Supplement 2. **eFigure 1.** Overview of the MUTTON-HF Intervention **eTable.** Additional Baseline Characteristics of the Study Cohort (N = 20) **eFigure 2.** Summary of Implementation Outcomes for MUTTON-HF Among Patients and Community Partners **eAppendix 1.** Baseline Patient Survey **eAppendix 2.** Patient Postintervention Survey **eAppendix 3.** Semistructured Interview Questions for Patients **eAppendix 4.** Semistructured Interview Questions for Farmers and Ranchers

Supplement 3.
Data Sharing Statement
