# Supplementary material for: Feasibility of an Indigenous Food Is Medicine Program for Patients With Heart Failure in Rural Navajo Nation: The MUTTON-HF Nonrandomized Clinical Trial
Source: JAMA Netw Open. 2026 Feb 6;9(2):e2556117. doi: 10.1001/jamanetworkopen.2025.56117 (PMC12881988; doi:10.1001/jamanetworkopen.2025.56117)
Supplement: Supplement 1. — Trial Protocol [file jamanetwopen-e2556117-s001.pdf]

PROTOCOL TITLE: MUTTON-HF (Medically **U**tilized **T**ailored **T**raditional food to **O**ptimize **N**utrition) in Heart Failure

Version Date 10/31/2024

Clinicaltrials.gov Registration: NCT06675331

SECTION 1: RESEARCH PLAN

Statement of Purpose:

The American Indian and Alaska Native population has experienced significant cardiovascular health disparities compared with other racial and ethnic groups in the U.S. [1] Heart failure, in particular, causes significant morbidity and mortality in Navajo Nation. For many Navajo patients, similar to other American Indian populations, food insecurity is a major driver of health disparities. [2][3] In fact, qualitative data from our heart failure patient advisory committee have found that 89% of patients with heart failure believe nutrition insecurity is a major barrier to optimal health. Dietary factors are believed to be an important cause of hospitalizations in patients with heart failure and cardiovascular outcomes. There is increasing evidence that direct dietary support, such as produce prescription or provision of medically tailored meals may improve cardiovascular outcomes and disease-specific quality of life.[4][5] Furthermore, there has been an increased focus in Indigenous communities to reclaim traditional indigenous foods to improve health. However, more evidence of the benefit of traditional Indigenous foods for cardiovascular health is needed.

We, therefore, in discussion with community members and tribal partners at two Indian Health Service (IHS) sites in Navajo Nation, will implement and evaluate the effectiveness of a medically and Native-sourced culturally tailored meal delivery program to improve outcomes in heart failure in rural Navajo Nation. This study will include two phases, with a phase I pilot feasibility study, followed by phase II—a comparativeness effectiveness randomized controlled trial to compare the implementation of our medically and culturally tailored meal delivery program compared to usual care. For phase I, we will conduct a one-arm pilot trial of the MUTTON-HF intervention to determine 1) feasibility of the intervention and outcome assessment and 2) acceptability of the intervention and 3) fidelity of the intervention. We will enroll 20 patients to receive medically tailored meals (14 meals weekly) for 30 days to inform the phase II comparativeness effectiveness trial.

Phase I outcomes will include implementation outcomes such as feasibility and acceptability of the intervention including the various delivery mechanisms and meals as measured by quantitative (i.e. % meals delivered and % meals received by patient) and qualitative methods, of outcome assessment including surveys and laboratory evaluation (% with completed outcome assessment), and fidelity of intervention as measured by quantitative (% meals consumed). We will additionally measure feasibility and acceptability of supporting local food systems by measuring % meals with locally sourced produce and meat, and % sourced from Navajo farmers and ranchers specifically. We will also explore implementation outcomes with community partners including farmers, growers, ranchers, food pantry.

Goal: Demonstrate feasibility and acceptability target can be achieved with ≥ 75% meals delivered to patient and ≥70% meals consumed as described below.

Probable Duration of Phase: 6 months

Research Plan: We plan to conduct a single arm pilot feasibility study. This will be done at two IHS clinical sites in the Gallup Service Unit at Indian Health Service: Gallup Indian Medical Center (GIMC) and Tohatchi Health Clinic (THC)

Study Population: Patients 18 years of age or older, with diagnosis of heart failure based on ICD code I50* with a hospitalization (any cause) in the last 12 months. Only patients with active prescription through IHS and engaged in care at our centers (clinical visit within last 12 months) will be included. We will exclude patients on hospice care or those residing in an acute care or skilled nursing facility, or any patient currently residing outside of the 50-mile IHS Gallup Service Unit catchment area.

Sample Size: We will enroll 20 patients for this pilot feasibility study

Subject Recruitment: Eligible patients are identified through a query through iCARE, a EHR based data system through the IHS EHR. Patients with ICD10 diagnostic criteria for HF will be identified (ICDI50*). The inclusion criteria will be all adults ≥18 years, who have a prescription in the IHS system in the last 12 months and have had a clinical visit at GIMC or THC in the last 12 months, as well as a hospitalization (all-cause) in the last 12 months. Patients will be consented verbally either at the health center or verbally over the phone to enroll.

Enrollment: All patients that meet eligibility criteria and consent to participate will be enrolled to receive meal delivery simultaneously; all enrolled patients will start receiving the intervention at the same starting time point, t=0.

Intervention

1. A. ***Designing Culturally Relevant Medically Tailored Meals***

We will partner with Tocabe, an Indigenous-run meal program, offering healthier and traditional foods, utilizing food from Native farmers and food producers.[6] Tocabe is a native-sourced and native-owned organization, which provides ready-made medically tailored meals to customers. In addition, they have extensive experience providing Indigenous and locally sourced foods direct to tribes. As above, meals will be designed to incorporate local and traditional Navajo foods. Meals will also be medically tailored to be consistent with sodium-restricted Dietary Approaches to Stop Hypertension as per American Heart Association recommendations.[6][7] Meals will also be additionally medically tailored for patients with type 2 diabetes and chronic kidney disease. Meals will be designed with cultural diet experts as well as

registered dieticians to ensure meals are optimized medically and culturally. As part of Phase I, we will have local experts in Diné cuisine meet with our partners at Tocabe, to help develop a menu of culturally relevant meals. As part of this process, we will determine key high priority Navajo food items, e.g. mutton, that are culturally important to include as part of our medically tailored meals. We will design different meal plans with a variety of options, all based on culturally relevant foods which will be incorporated as part of our medically tailored plan. As much is feasible, we will partner with local Navajo farmers coalitions to locally source all ingredients and produce, not only to strengthen local food systems, but to also increase to increase accessibility of the meals and the likelihood of long-term adoption of recipes and meals at home after the intervention period.

1.B *Supply Chain and Meal Delivery Logistics*

Phase I will also include establishing and securing a supply chain for meals, and securing logistics for meal delivery, particularly for those living rurally on the reservation. Among a sample of n=22 HF patients, we found that 95% of patients had PO boxes (without a physical address/mailbox). Given this, we have partnered with the Gallup Food Pantry, and will have frozen meals prepared, packaged, and delivered to the Gallup Food Pantry in bulk as a central food hub, with subsequent patient distribution. Patients that live locally and are able to pick up meals will come pick up meals weekly. Those who are unable to pick up meals (or do not come for their meals), will have meals delivered weekly by the food pantry's mobile unit. For those without refrigeration, meals will be delivered to local chapter houses, and patients will pick up 3-day supply of meals in freezer boxes. In addition to refrigeration, we will also assess and address household infrastructure (e.g. ability to store and heat meals). We will develop these logistical plans as part of phase I, and then will be evaluating them in this pilot feasibility trial.

1. C. Pilot Feasibility Trial

Prior to trial enrollment, we will conduct a 6-month feasibility trial in which we enroll a total of 20 patients, to test feasibility of home delivery of meals and obtain patient input on acceptability to optimize our larger scale trial. Our IHS study facilities have a catchment area of a 50-mile radius. Of 22 sample patients, selected at random from our EHR with heart failure, patient's locations of residence are shown in the map.

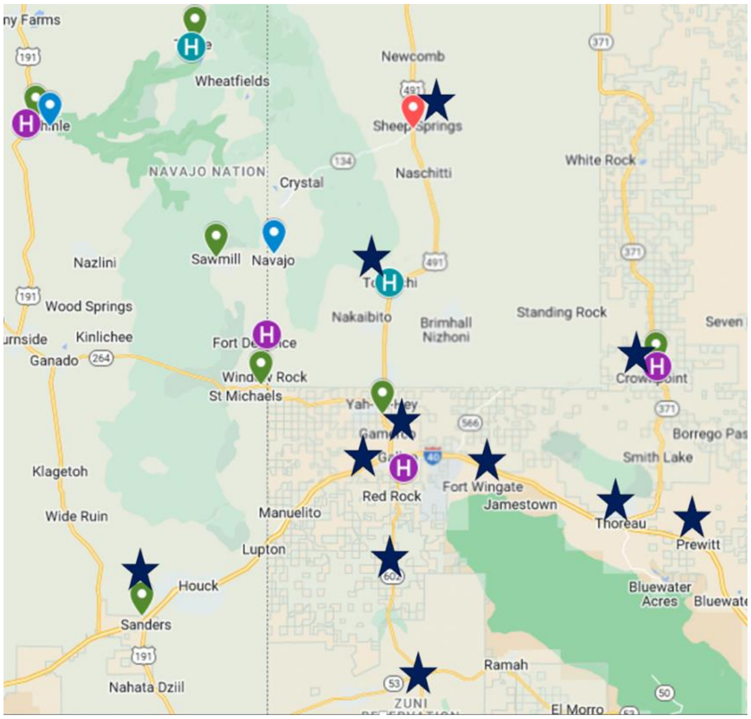

Given the geographic distance between patients, the rurality of locations, and lack of mailboxes or mailing addresses for many patients, we will conduct a pilot feasibility study of 20 patients to ensure feasibility of patient delivery and acceptability of the medically and culturally tailored meals. Patients will receive 2 meals daily (14 meals weekly) for 4 weeks. Meals will be prepared, packaged, and delivered in bulk to our central food hub (Gallup Food Pantry).

From there, based on patient preference, a 1-week supply of meals will be either picked up weekly by the patient or delivered by the food pantry's mobile unit to patient's local chapter house, closest clinic site (either GIMC or THC), or their home based on patient preference and feasibility based on geographic location. Patients will also receive 1 cooking class to learn how to make culturally relevant recipes from the meal plan, all incorporating locally available ingredients. At baseline, we will assess patient's dietary preferences and restrictions.

Baseline Characteristics: In addition to the American Heart Association sociodemographic core measures, we will also collect tribal affiliation, language, and in which local chapter/community patients reside.

Outcomes:

Primary outcomes: primary outcomes will be implementation outcomes to assess feasibility and acceptability of the intervention with both quantitative and qualitative assessment as described below.

Feasibility and acceptability outcomes:

Feasibility of the intervention

- We will assess feasibility of the intervention quantitatively by evaluating the following
    - Percentage of meals delivered successfully to the Gallup Food Pantry
    - Percentage of meals successfully received by the patient
    - We will also assess for differences in successful delivery between different delivery mechanisms (i.e. pick up at food pantry, delivery to mini-hub [I.e. Chapter House], peer-delivery (one member delivers to other enrolled patients in same community, and community health worker delivery), and identify any delivery mechanisms that were unsuccessful to inform phase II. This will help us determine optimal appropriate delivery strategies for different community and patient situations.
    - We will survey patients post-intervention with question to assess feasibility: "I would rate getting the meals (either picking up or delivery): Very easy, Somewhat easy, Neither easy nor difficult, Somewhat difficult, Very difficult.

Acceptability of the Intervention

- We will evaluate acceptability of the intervention among the patients through quantitative assessment using the validated acceptability of the intervention (AIM) measure.[8]
- We will also assess acceptability of the intervention through assess the Net Promoter Score (i.e. how likely is it that you would recommend this program to a community member?").
- Patients will also rate meals from 1-5 and the amount of the meal they consumed (none [or only a few bites]
- Patients will also be surveyed post-intervention the following questions to assess acceptability:
    - How would you rate the meals overall, in terms of taste (excellent, good, average, below average, bad?"

We will also perform semi-structured interviews of patients to assess feasibility and acceptability through qualitative methods. Semi-structured interviews will be guided by the Consolidated Framework

for Implementation Research (CFIR), and we will explore multiple constructs within each CFIR domain that are hypothesized by the study team and based on existing literature to be relevant to acceptability and feasibility of the program.[9]

Secondary Outcomes:

Feasibility and Acceptability of Supporting Local Food Systems

- We will assess feasibility among our community partners by assessing the validated feasibility of the intervention measure (FIM) [8] among our food pantry partners.
- We will also assess the % of meals that include locally sourced ingredients, % of meals sourced by Native suppliers/farmers (produce), % of meals soured by Native ranchers (meat)
- We will assess feasibility and acceptability among our community partners by performing semi-structured interviews of non-patient stakeholders (i.e. farmers that supplied produce, livestock farmers, food pantry) to assess acceptability and feasibility of providing produce for the meals, as well as the degree to which the program contributed to strengthening the local food system. Semi-structured interviews will be guided by the Consolidated Framework for Implementation Research (CFIR), and we will explore multiple constructs within each CFIR domain that are hypothesized by the study team and based on existing literature to be relevant to acceptability and feasibility of the program.[9]

Feasibility of Outcome Assessment

- We will determine the proportion of patients that completes baseline, as well as post-implementation survey and laboratory data.
    - We will consider ≥ 80% completion rates as a success.

Fidelity

- We will assess fidelity of the intervention by evaluating:
    - We will have patients' complete food diaries for 3 days during week 1 and week 4 of the intervention.
    - % of meals consumed: Patients will also fill out meal cards weekly with percent consumed (None [or a few bites], ¼, ½, ¾, all [or nearly all]).
        - We will stratify meals consumed by whether the meal used traditional Diné ingredients or not.
    - We will additionally survey participants post-intervention the following:
        - In terms of leftovers, how much of the meals were not eaten and had to be thrown out? (all, most, about half, less than half, none).
- We will consider ≥ 70% meals consumed (at least ½ or more of the meal) or ≥70% indicating they ate "most" or more of the meals as a success.

Adoption

- We will survey participants post-intervention the following using a Likert scale:
    - How likely is it that this program will change your diet to be healthier moving forward?
    - How likely is it that you will add any of the meals or recipes from this program to your on-going diet in the future?

We will also pilot outcome assessment for the larger phase II comparativeness effectiveness trial. We will also evaluate the primary outcome of phase II of ER visit or hospitalization within 90 days and will pilot collection of key secondary outcomes of phase II including laboratory biomarker data (comprehensive metabolic panel, lipid panel, HbA1c, albumin, prealbumin, NT-proBNP), blood pressure, weight, body mass index, Kansas City Cardiomyopathy Score (and its components including summary score, physical limitation score, symptom frequency score, quality of life score and social limitation score)[10], diet quality using the 10-item DSQ (with additional question assesses intake of traditional Diné foods)[11], food security (utilizing the USDA 6-item food security survey)[12], and cultural connectiveness score at baseline and 30-days post intervention. Lab work will be permitted to be collected 14 days before 60 days mark and up to 30 days after (from 46 days-90 days post randomization).

Additional details on pilot collection of key secondary outcome measures for phase II:

Diet Quality

- We will assess diet quality using the 10-item DSQ [11] with an addition question to assess traditional Dine food intake (*During the past month, how often did you eat traditional Diné foods (such as blue corn mush, steamed, roasted or dried corn, sumac berries, mutton, local varieties of squash or beans)* at baseline and at the end of the intervention.

Food Security

- We will also evaluate baseline and 60-day rates of food insecurity utilizing the USDA Adult Food Security: Six Item short Form survey (USA FSSM 6-Item).[12]

Indigenous Cultural Connectedness:

- We will also measure cultural connectedness utilizing a 6-question modified version of the validated Cultural Connectedness Scale-California (CCS-CA) at baseline and at 60 days.[13] This is a validated score, developed by Indigenous scholars to measure cultural connectiveness in Indigenous populations, and has been shown to correlate with physical health and be a social determinant of health.[14] This scale has previously been modified for use in Diné (Navajo) populations specifically by our group.

Exploratory Outcomes:

Additional exploratory outcomes will prescription rates for all medication as well as guideline-directed medical therapy specifically (for heart failure with reduced ejection fraction this would include ACEi/ARB/ARNI, Beta-blocker, SGLT2 inhibitors, and mineralocorticoid receptor antagonist; for HFpEF this would include SGLT2 inhibitors).

Physical Activity

- We will evaluate how much physical activity/exercise patients are participating in weekly at baseline, and at 30 days by asking patients to estimate the number of minutes weekly that they are participating in formal exercise or physical activity.

General Health Status:

- We will evaluate patients' general health status using a single general health status question (would you say that in general your health is excellent, very good, good, fair, or poor) at baseline and post-intervention

Outcomes determined by review of medical, hospital, and billing records, as well as patient surveys and semi-structured interviews. We will extract medication at baseline and every 30 days during the study period to assess for changes in medical therapy. All patients will be provided a scale for weight measurements. Patients will be instructed to take weights first thing in the morning (or pick a time-up front for all weight measurements for consistency). All patient-reported measures, including KCCQ and CCS-CA scores will be over the phone. Therefore, patients will only need to come in for lab work at baseline and at 30 days. For patients who are unable to come with for lab work at 30 days (and within the window of 15-60 days), then we will have public health nurses provide outreach to collect blood work in the home. Patients will receive a text message (if they have a cell phone) or phone call on the landline (if no cell phone) to remind them to complete food diaries, weights, and other patient-reported measures in a timely manner. For those that have trouble completing written food diaries, we will collect this information verbally over the phone.

For subsequent planning purposes, we will also track costing data to determine the complete cost of the intervention and estimate total program costs (including all resources, even donated, involved in the implementation of the program, but not including research activities such as blood tests).

Informing phase II: We will consider pilot feasibility trial a success if ≥75% of meals delivered to/received by the patient, and ≥ 70% meals consumed as described below. Results from the analyses will inform phase II comparativeness effectiveness trial of the intervention.

Intervention Duration: The study team will implement the food delivery model for 30 days.

Inclusion/Exclusion Criteria

Inclusion

- Age ≥18 years
- ICD I50* diagnosis
- Clinical encounter in last 12 months
- Prescription in IHS system in past 12 months
- Primary care physician at one of the two IHS sites
- Hospitalization (any cause) in the past 12 months

Exclusion

- Hospice care
- Living in acute rehabilitation or skilled nursing facility

- Living outside the Gallup Service Unit (outside 50-mile catchment area)

How will eligibility be determined, and by whom?

- Eligibility of patients will be assessed by the study team based on the patient's medical record.

Methods to Reduce Loss to Follow-up: Patients that do not pick up meals in the planned mechanism within 2 days of planned pick up, will have meals delivered to them at home by a CHR. If patients are not participating in the study as planned, they will be contacted by phone. If unable to reach by phone x 3 attempts, family members will be contacted. If unable to reach anyone, then we will have a public health nurse/community health representative conduct a home visit.

Timeline for Phase I and Phase II

	YEAR 1												YEAR 2											
	M1	M2	M3	M4	M5	M6	M7	M8	M9	M10	M11	M12	M13	M14	M15	M16	M17	M18	M19	M20	M21	M22	M23	M24
Phase I: Meal Curation of Traditional Navajo Meals	X	X	X																					
Phase I: Secure supply chain	X	X	X																					
Phase I: Secure local suppliers for produce and livestock		X	X	X																				
Eastern Navajo Agency Council Approval			X	X	X																			
IRB Approval				X	X																			
Phase I: Pilot feasibility (n=20)					X	X																		
Phase I: Pilot feasibility outcome assessment						X	X																	
Phase II: Trial																								
Determine eligible sample in our healthsystem		X	X	X	X	X	X																	
Enrollment								X	X															
25% Sample Size (n=50)									X	X														
50% Sample Size (n=100)										X	X													
75% Sample Size (n=150)											X	X												
100% Sample Size (n=200)												X	X											
Study Period-Primary Outcome																X								
Study Period-ALL Outcome Assessments Ends																			X					
Data Analyses																			X	X	X			
Present to Stakeholders and Tribal Council																					X	X	X	
Dissemination																						X	X	X

In-Kind Funding

- **The Indian Health Service** – The IHS will fund all patient scales, allow for all lab work to occur at IHS facilities free of charge, and provide funding for patient navigators and heart failure nurses to assist in tracking patients, calling patients to remind them of lab work, assisting with completion of patient-reported outcomes/surveys and case report forms for trial outcomes.
- **Dr. Eberly** with funding through the **Robert A. Winn Diversity in Clinical Trial Career Development Award** will fund all clinical trial support through the clinical research collaboration unit at Penn.
- **Tocabe Inc**- will donate recipe cards of the meals provided, as well as the recipe cards for the control group.
- **The Gallup Food Pantry-** will donate their freezer space and have agreed to serve as the central food hub to meal delivery and will provide their services for decentralized patient meal delivery including their mobile delivery unit as needed.

Additional Stakeholders and Partners

- **Navajo Area Department of Health**- Dr. Paula Mora, who serves as the Director for the Navajo Area Department of Health for the IHS, is fully supportive of this study and will serve on our community advisory board. She works closely with the Navajo Nation Department of Health, who are also supportive of this work. We will be working closely with the Navajo Nation Department of Health on this study so that if our program is effective, we can leverage our partnerships with tribal leaders to secure pathways to allow for sustainable funding and adoption of this program long-term.
- **Indian Health Service Headquarters Office of Quality:** Our team includes Dr. Benjamin Feliciano, improvement advisor, and Dr. Philippe Champagne, director of quality improvement for the IHS HQ Office of Quality Division of Innovation. This study is supported by an Indian Health Service Innovations Award, which provides logistical support from the IHS Office of Quality. Additionally, as part of an IHS innovations award, the results of this study may inform adoption and expansion more broadly throughout the IHS of such programs.
- **Indian Health Service Headquarters Food is Medicine Coalition:** Our community advisory board includes Dr. Stacy Hammer who is the principal nutrition consult for the IHS nationally and serves as the IHS representative for the federal agencies' Food is Medicine Coalition. These results will be critical to allow the coalition to advocate for increased congressional funding to support food is medicine initiatives throughout the HIS nationally.
- **Shiprock Farmers Coalition/Local Navajo Farmers:** To locally source ingredients for our meals with Tocabe, we will be partnering with local farmers coalitions such as the Shiprock Farmers Coalition and other local produce and livestock farmers to support local economic development and support local food systems.

Community Advisory Board

We have established a community advisory board of Our community advisory board will include the following individuals to ensure not only success of the proposed research project, but also to allow for sustainability of the program if found to be effective:

- Dr. Paula Mora, the Director of Navajo Area Department of Health
- Stacy Hammer, RD- Principal nutrition consult for the IHS Headquarters, IHS representative for the federal agencies' Food is Medicine Coalition
- Sharon Sandman- Community-based partner leading initiatives to support local farmers and food sovereignty throughout Navajo Nation
- Colleen Biakeddy - Diné (Navajo) sheep rancher, expert in traditional Navajo sheep herding. She will help locally source USDA approved Mutton for meal delivery and to support local livestock farmers as well as ensure traditional and ethical use of sheep for meals, as well as provide expertise on experience of challenges facing local farmers to source produce and meat.
- Commander Pamela Detsoi-Smiley: Chief Executive Officer of Gallup Indian Medical Center of the Indian Health Service
- Rory Aufderheide-Primary care physician at one of the IHS sites (THC), on the Board of Directors for Gallup Food Pantry with expertise in food is medicine initiatives locally.
- Patient and caregiver- we will invite at least 1-2 patients and caregivers from our previously implemented patient advisory heart failure board to join our community advisory board for this study.

We will meet monthly with the community advisory board during the planning stages, and then bimonthly during implementation to center community perspectives and assess implementation outcomes and feedback, followed by quarterly meetings after the active implementation phases.

Protection of Human Subjects: Protection of Human Subjects:

This study is aimed at implementing a model to improve access to healthy, nutritious foods for patients. Therefore, this poses a minimal risk to patients. There is prior literature that suggests that medically tailored meal delivery improves quality of life, including Kansas City Cardiomyopathy Questionnaire summary score, as well as potentially a trend to lower heart failure hospitalizations, without significant diet-related adverse events.[4]

However, there are no prior studies of medically and culturally tailored meals in this population. However, we will be medically tailoring meals to meet guideline recommendations. [6][7]. Meals will be designed with registered dietician input to ensure safety is optimized. Our Data Safety Monitoring committee will perform an interim analysis of the data after 60 days post randomization.

Human subjects' involvement, characteristics, and design: The studies outlined in this proposal depend on the enrollment of individuals with heart failure. No vulnerable populations are being specifically targeted. We are limiting enrollment to individuals above age 18 years as the etiology and practices surrounding heart failure in pediatrics populations differ significantly from those in adults. All data is transmitted in encrypted and secure fashion, stored on servers with "triple-lock" certification, and is available only to members of the study team, IRB, and any state or federal agencies with auditing power.

Sources of Materials: No biological materials will be obtained or stored as part of these studies. Only data, as collected during set time points from the EHR will be obtained. Data includes medical record elements such as demographics, pharmacy records such as medication prescription and dosing, laboratory values, and administrative codes. All data will be stored without PHI. However, we will retain a linking dataset to be able to re-link individual data to actual patients for future studies and ongoing efforts through the HIS. Access to individually identifiable information will be limited to the PI of the study, and only then via a linking file as aforementioned. All data used for analysis and dissemination to other investigators will be de-identified.

Over or under treatment: Providers will be blinded to patient treatment assignment and thus it is unlikely that treatment will differ between patients. However, we will also be extracting medication changes during the study period to ensure there were no significant differences in medical therapy treatment between the two groups.

Potential benefits of the proposed research to the subjects and others: Subjects in this study may directly benefit from receiving healthier food options for their care. In addition, this may improve their knowledge on healthy and traditional foods and dietary practices that may be sustained after the duration of the study. Additionally, the results of these studies may lead to significant benefit in the IHS and other rural Indigenous communities. This model could be similarly expanded to other sites. The risk/benefit ratio, given the minimal risk to study subjects, is more than acceptable in this series of studies.

Data and Safety Monitoring Plan:

• What is the investigator’s assessment of the overall risk level for subjects participating in this study?

We believe that this poses minimal risk to the patients.

• If children are involved, what is the investigator’s assessment of the overall risk level for the children participating in this study? No children will be involved.

This study poses **i. Minimal risk** ii. Greater than minimal

The principal investigators (PI) is responsible for monitoring the data, assuring protocol compliance, and conducting the safety reviews at the specified frequency regularly. During the review process the PIs will evaluate whether the study should continue unchanged, require modification/amendment, or close to enrollment. The PIs or the Navajo Nation Human Research Review Board (Navajo Nation IRB) have the authority to stop or suspend the study or require modifications.

This protocol presents minimal risks to the subjects and Unanticipated Problems Involving Risks to Subjects or Others (UPIRSOs), including adverse events, are not anticipated. In the unlikely event that such events occur, Reportable Events (which are events that are serious or life-threatening and unanticipated (or anticipated but occurring with a greater frequency than expected) and possibly, probably, or definitely related) or Unanticipated Problems Involving Risks to Subjects or Others that may require a temporary or permanent interruption of study activities will be reported immediately (if possible), followed by a written report within 5 calendar days of the Principal Investigator becoming aware of the event to the IRB (using the appropriate forms from the website) and any appropriate funding and regulatory agencies. The investigator will apprise fellow investigators and study personnel of all UPIRSOs and adverse events that occur during the conduct of this research project through regular study meetings and via email as they are reviewed by the principal investigator.

Statistical Considerations

Sample Size: We will pilot this in 20 patients who meet inclusion criteria, with purposeful selection for a variety of communities, Chapter Houses, ages and sexes to ensure broad representation across the catchment area to identify patient and community specific challenges and facilitators for implementation.

Interim Analysis: None

Statistical Analysis:

We will determine descriptive statistics of baseline characteristics of participants. Descriptive output will be generated for semi-quantitative responses to survey questions, including % of responses. We will assess differences from baseline to week 4 changes in continuous outcomes including laboratory evaluation and biomarkers, food security score, CCS, and KCCQ score using paired t-test, and proportions using McNemars test.

Semi structured interviews will be audio-recorded and transcribed for thematic analysis. Transcripts will be analyzed using an integrated approach, an iterative process to determine themes and patterns present in the data.[15] The interviewers will develop the initial codebook together, and then will independently dual code subsets of the transcripts to identify common themes and iteratively develop a

final codebook, with periodic assessment of inter-rater reliability. We will follow the consolidated criteria for reporting qualitative research (COREQ) guidelines.[16]

References

1. Eberly LA, Shultz K, Merino M, Brueckner MY, Benally E, Tennison A, Biggs S, Hardie L, Tian Y, Nathan AS, Khatana SAM, Shea JA, Lewis E, Bukhman G, Shin S, Groeneveld PW. Cardiovascular Disease Burden and Outcomes Among American Indian and Alaska Native Medicare Beneficiaries. JAMA Netw Open. 2023 Sep 5;6(9):e2334923. doi: 10.1001/jamanetworkopen.2023.34923. PMID: 37738051; PMCID: PMC10517375.
2. Hutchinson RN, Shin S. Systematic review of health disparities for cardiovascular diseases and associated factors among American Indian and Alaska Native populations. PLoS One. 2014 Jan 15;9(1):e80973. doi: 10.1371/journal.pone.0080973. PMID: 24454685; PMCID: PMC3893081.
3. Berryhill K, Hale J, Chase B, Clark L, He J, Daley CM. Food security and diet among American Indians in the Midwest. J Community Health. 2018;43(5):901–7.
4. Hummel SL, Karmally W, Gillespie BW, Helmke S, Teruya S, Wells J, Trumble E, Jimenez O, Marolt C, Wessler JD, Cornellier ML, Maurer MS. Home-Delivered Meals Postdischarge From Heart Failure Hospitalization. Circ Heart Fail. 2018 Aug;11(8):e004886. doi: 10.1161/CIRCHEARTFAILURE.117.004886. PMID: 30354562; PMCID: PMC6205816.
5. Hager K, Du M, Li Z, Mozaffarian D, Chui K, Shi P, Ling B, Cash SB, Folta SC, Zhang FF. Impact of Produce Prescriptions on Diet, Food Security, and Cardiometabolic Health Outcomes: A Multisite Evaluation of 9 Produce Prescription Programs in the United States. Circ Cardiovasc Qual Outcomes. 2023 Sep;16(9):e009520. doi: 10.1161/CIRCOUTCOMES.122.009520. Epub 2023 Aug 29. PMID: 37641928; PMCID: PMC10529680.
6. Eckel RH, Jakicic JM, Ard JD, Hubbard VS, de Jesus JM, Lee I-M, Lichtenstein AH, Loria CM, Millen BE, Miller NH, Nonas CA, Sacks FM, Smith SC, Svetkey LP, Wadden TW, Yanovski SZ. 2013 AHA/ACC Guideline on lifestyle management to reduce cardiovascular risk. A report of the American College of Cardiology/American Heart Association Task Force on Practice Guidelines.Circulation. 2013; 129(25 suppl 2):S76–S99. doi: 10.1161/01.cir.0000437740.48606.d1
7. Appel LJ, Frohlich ED, Hall JE, Pearson TA, Sacco RL, Seals DR, Sacks FM, Smith SC, Vafiadis DK, Van Horn LV. The importance of population-wide sodium reduction as a means to prevent cardiovascular disease and stroke: a call to action from the American Heart Association.Circulation. 2011; 123:1138–1143. doi: 10.1161/CIR.0b013e31820d0793
8. Weiner, B. J., Lewis, C. C., Stanick, C., Powell, B. J., Dorsey, C. N., Clary, A. S., ... & Halko, H. (2017). Psychometric assessment of three newly developed implementation outcome measures. Implementation Science, 12(1), 108.
9. Damschroder LJ, Aron DC, Keith RE, Kirsh SR, Alexander JA, Lowery JC. Fostering implementation of health services research findings into practice: a consolidated framework for advancing implementation science. Implement Sci 2009;4:50.
10. Green CP, Porter CB, Bresnahan DR, Spertus JA. Development and evaluation of the Kansas City Cardiomyopathy Questionnaire: a new health status measure for heart failure. J Am Coll Cardiol. 2000 Apr;35(5):1245-55. doi: 10.1016/s0735-1097(00)00531-3. PMID: 10758967.
11. Dietary Screener Questionnaire (DSQ) 1- item scale. dsq-quick-guide_11-2023.pdf (nutritionincentivehub.org). Accessed June 9th 2024.
12. U.S. Household Food Security Survey Module: Six-Item Short Form Economic Research Service, USDA September 2012 Six-item Short Form Food Security Survey Module (usda.gov) Accessed 6/9/24.

13. Snowshoe A, Crooks CV, Tremblay PF, Craig WM, Hinson RE. Development of a cultural connectedness scale for first nations youth. Psychol Assess. 2015;27(1):249. https://doi.org/10.1037/a0037867.
14. Masotti P, Dennem J, Bañuelos K, Seneca C, Valerio-Leonce G, Inong CT, King J. The Culture is Prevention Project: measuring cultural connectedness and providing evidence that culture is a social determinant of health for Native Americans. BMC Public Health. 2023 Apr 21;23(1):741. doi: 10.1186/s12889-023-15587-x. PMID: 37085784; PMCID: PMC10120477.
15. C U. Grounded theory for qualitative research: A practical guide. Thousand Oaks, CA: SAGE Publications; 2013.
16. Tong A, Sainsbury P, Craig J. Consolidated criteria for reporting qualitative research (COREQ): a 32-item checklist for interviews and focus groups. Int J Qual Health Care 2007;19:349-57.
