# Supplementary material for: Feasibility of an Indigenous Food Is Medicine Program for Patients With Heart Failure in Rural Navajo Nation: The MUTTON-HF Nonrandomized Clinical Trial
Source: JAMA Netw Open. 2026 Feb 6;9(2):e2556117. doi: 10.1001/jamanetworkopen.2025.56117 (PMC12881988; doi:10.1001/jamanetworkopen.2025.56117)
Supplement: Supplement 2. — eFigure 1. Overview of the MUTTON-HF Intervention eTable. Additional Baseline Characteristics of the Study Cohort (N = 20) eFigure 2. Summary of Implementation Outcomes for MUTTON-HF Among Patients and Community Partners eAppendix 1. Baseline Patient Survey eAppendix 2. Patient Postintervention Survey eAppendix 3. Semistructured Interview Questions for Patients eAppendix 4. Semistructured Interview Questions for Farmers and Ranchers [file jamanetwopen-e2556117-s002.pdf]

Supplemental Online Content

Eberly L, George C, Sandman S, et al. Feasibility of an Indigenous Food Is Medicine program for patients with heart failure in rural Navajo Nation: the MUTTON-HF nonrandomized clinical trial. *JAMA Netw Open*. 2026;9(2):e2556117. doi:10.1001/jamanetworkopen.2025.56117

eFigure 1. Overview of the MUTTON-HF Intervention
eTable. Additional Baseline Characteristics of the Study Cohort (N = 20)
eFigure 2. Summary of Implementation Outcomes for MUTTON-HF Among Patients and Community Partners
eAppendix 1. Baseline Patient Survey
eAppendix 2. Patient Postintervention Survey
eAppendix 3. Semistructured Interview Questions for Patients
eAppendix 4. Semistructured Interview Questions for Farmers and Ranchers

This supplemental material has been provided by the authors to give readers additional information about their work.

© 2026 Eberly L et al. *JAMA Network Open*.

eFigure 1. Overview of the MUTTON-HF Intervention

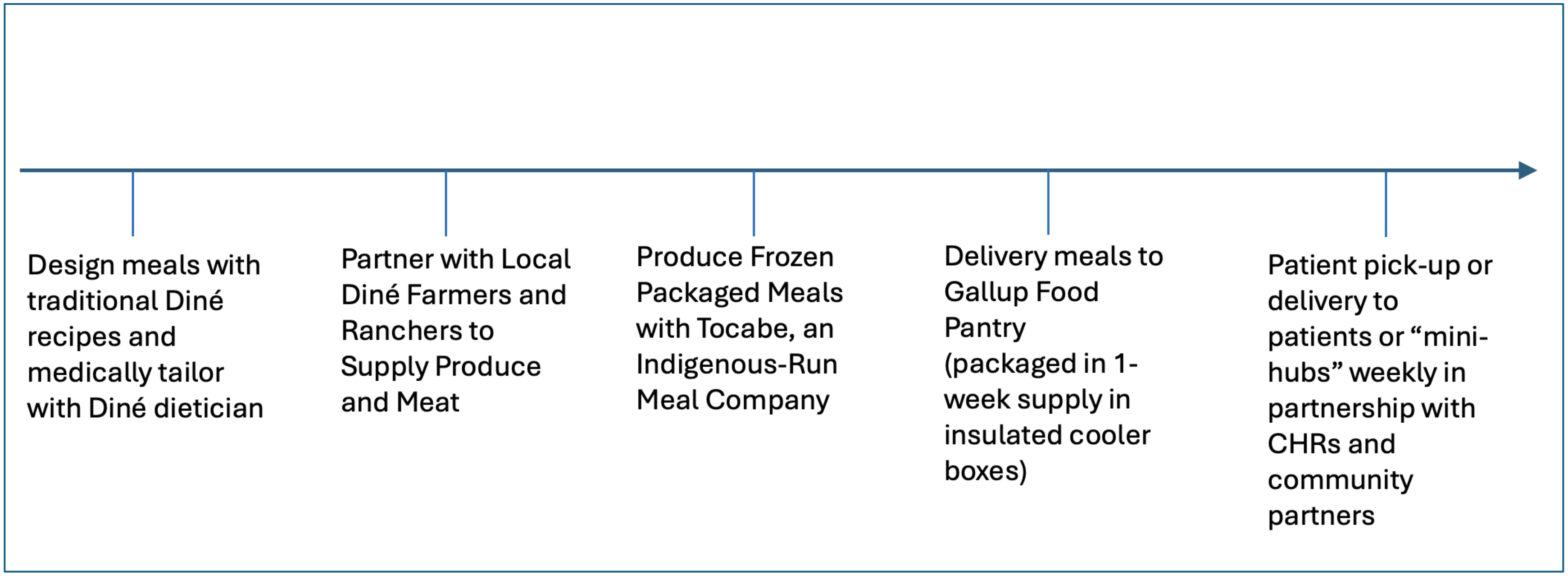

© 2026 Eberly L et al. *JAMA Network Open*.

eTable. Additional Baseline Characteristics of the Study Cohort (N = 20)

Medications prescribed, n (%)	
SGLT2i	18 (90%)
Metformin	3 (15%)
Beta-blocker	19 (95%)
ACEi/ARNI/ARNI	19 (95%)
Loop diuretic	11 (55%)
Thiazide diuretic	0 (0%)
Insulin	1 (5%)
MRA	15 (75%)
Statin	17 (85%)
Ezetimibe	0 (0%)
PSCK9 inhibitor	1 (5%)
GLP1RA	11 (55%)
Amlodipine	0 (0%)
Sulfonylurea	1 (5%)
Another BP agent	3 (15%)
Fish Oil	0 (0%)

SGLT2, sodium–glucose cotransporter 2 inhibitors; ACEi, angiotensin-converting enzyme inhibitor; ARB, angiotensin receptor blockers; ARNI, angiotensin receptor/neprilysin inhibitor; MRA-mineralocorticoid receptor antagonist; PCSK9-I, *Proprotein convertase subtilisin/kexin type 9 inhibitor;* GLP-1RA, glucagon-*like peptide-1 agonists*.

© 2026 Eberly L et al. *JAMA Network Open*.

eFigure 2. Summary of Implementation Outcomes for MUTTON-HF Among Patients and Community Partners

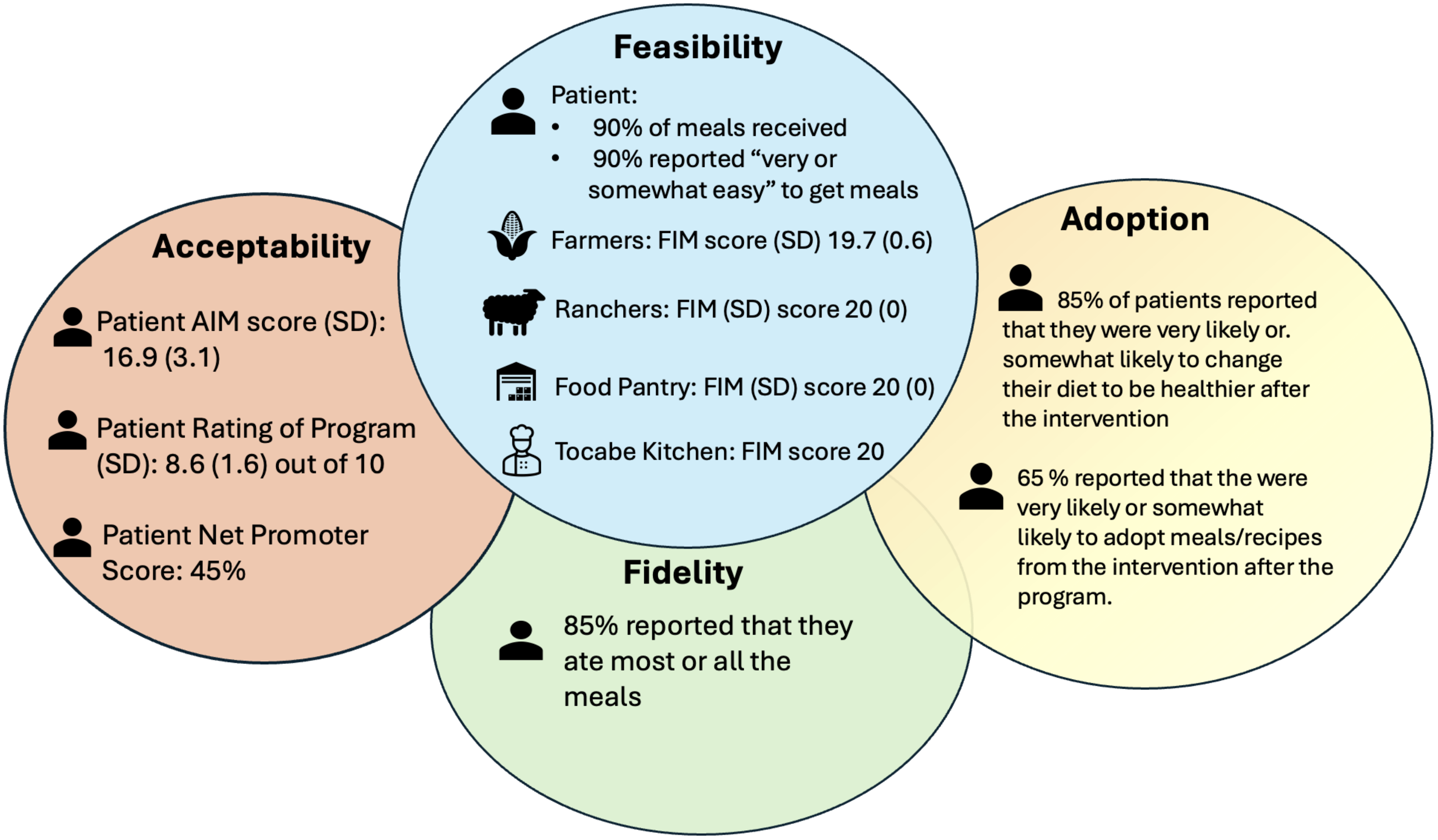

© 2026 Eberly L et al. *JAMA Network Open*.

eAppendix 1. Baseline Patient Survey

Participant ID ______________________ Date ____

Who filled out this form______________________________
How was form filled out: [ ] over the phone [ ] in person

Section A: Information about participant and household

1. What is your birthdate (month and year): ___/______

2. Your gender (Circle one)

- ☐ Male
- ☐ Female
- ☐ Transgender
- ☐ Non-binary / Two-spirit
- ☐ None of these describe me
- ☐ Prefer not to answer

3.

How would you describe yourself (mark all):

- ☐ American Indian/Alaska Native → Tribal affiliation(s): ______________
- ☐ White
- ☐ Hispanic or Latino/a
- ☐ Black or African American
- ☐ Asian
- ☐ Native Hawaiian or Pacific Islander
- ☐ Other: _________________
- ☐ Prefer not to answer / not sure

4. What Chapter or community do you live in?
____

5. Please mark state of residence

   1. AZ, NM

6. How many people live in your household? _______________

   1. How many adults?_______How many children?______________

7. Are you responsible for the cooking? [ ] Yes [ ] No [ ] Some of it

8. What is the best phone number to reach you? ___________________________________

   1. Do you have a secondary phone number (can be of family member if case we can't reach you)?_______________________________

9. Do you prefer phone call or text message?

   1. [ ] Phone
   2. [ ] Text

10. We'd like to know about your language preferences:

How well would you say you **speak** English?	☐ Very well	☐ Well	☐ Not well	☐ Not at all	☐ Prefer not to answer/not sure
How well would you say you **speak** Navajo?	☐ Very well	☐ Well	☐ Not well	☐ Not at all	☐ Prefer not to answer/not sure
How well would you say you **read** English?	☐ Very well	☐ Well	☐ Not well	☐ Not at all	☐ Prefer not to answer/not sure
How well would you say you **read** Navajo?	☐ Very well	☐ Well	☐ Not well	☐ Not at all	☐ Prefer not to answer/not sure

© 2026 Eberly L et al. *JAMA Network Open*.

CCS-S

How strongly do you agree with these statements?	Strongly agree	Agree	Don't agree or disagree	Disagree	Strongly disagree
In certain situations, I believe things like animals and rocks have a spirit like Diné people	☐	☐	☐	☐	☐
I plan to learn more about my Diné culture, such as its history, traditions and customs	☐	☐	☐	☐	☐
I have a traditional person or Elder who I talk to about Diné beliefs and culture	☐	☐	☐	☐	☐
I have spent time trying to find out more about being Navajo, such as my history, traditions and customs	☐	☐	☐	☐	☐
I feel like I belong to the Diné community	☐	☐	☐	☐	☐
I feel a strong attachment towards my Diné community	☐	☐	☐	☐	☐
The eagle feather has a lot of meaning to me	☐	☐	☐	☐	☐
I use mountain tobacco for guidance	☐	☐	☐	☐	☐
How many of your clans can you name?	☐ 1	☐ 2	☐ 3	☐ 4	☐ Not sure
How often does someone in your family or someone you are close with use sage, cedar or other Diné traditional herbs?	☐ Every day	☐ Every week	☐ Every month	☐ 1-2 times a year	☐ Never

Health questions

11. Would you say that in general your health is excellent, very good, good, fair, or poor?

[ ] Excellent [ ] Very Good [ ] Good [ ] Fair [ ] Poor

12. How many minutes would you estimate you exercise weekly?_________________

© 2026 Eberly L et al. *JAMA Network Open*.

13. Kansas City Cardiomyopathy Questionnaire (KCCQ-12)

Kansas City Cardiomyopathy Questionnaire (KCCQ-12)

The following questions refer to your **heart failure** and how it may affect your life. Please read and complete the following questions. There are no right or wrong answers. Please mark the answer that best applies to you.

1. **Heart failure** affects different people in different ways. Some feel shortness of breath while others feel fatigue. Please indicate how much you are limited by **heart failure** (shortness of breath or fatigue) in your ability to do the following activities over the past 2 weeks.

**Activity**	**Extremely** Limited	**Quite a bit** Limited	**Moderately** Limited	**Slightly** Limited	**Not at all** Limited	Limited for other reasons or did not do the activity
a. Showering/bathing	O	O	O	O	O	O
b. Walking 1 block on level ground	O	O	O	O	O	O
c. Hurrying or jogging (as if to catch a bus)	O	O	O	O	O	O
	1	2	3	4	5	6

2. Over the past 2 weeks, how many times did you have **swelling** in your feet, ankles or legs when you woke up in the morning?

Every morning	3 or more times per week but not every day	1-2 times per week	Less than once a week	Never over the past 2 weeks
O	O	O	O	O
1	2	3	4	5

3. Over the past 2 weeks, on average, how many times has **fatigue** limited your ability to do what you wanted?

All of the time	Several times per day	At least once a day	3 or more times per week but not every day	1-2 times per week	Less than once a week	Never over the past 2 weeks
O	O	O	O	O	O	O
1	2	3	4	5	6	7

4. Over the past 2 weeks, on average, how many times has **shortness of breath** limited your ability to do what you wanted?

All of the time	Several times per day	At least once a day	3 or more times per week but not every day	1-2 times per week	Less than once a week	Never over the past 2 weeks
O	O	O	O	O	O	O
1	2	3	4	5	6	7

5. Over the past 2 weeks, on average, how many times have you been forced to sleep sitting up in a chair or with at least 3 pillows to prop you up because of **shortness of breath**?

Every night	3 or more times per week but not every day	1-2 times per week	Less than once a week	Never over the past 2 weeks
O	O	O	O	O
1	2	3	4	5

[illegible]

© 2026 Eberly L et al. *JAMA Network Open*.

6. Over the past 2 weeks, how much has your **heart failure** limited your enjoyment of life?

It has **extremely** limited my enjoyment of life	It has limited my enjoyment of life **quite a bit**	It has **moderately** limited my enjoyment of life	It has **slightly** limited my enjoyment of life	It has **not limited** my enjoyment of life at all
O	O	O	O	O
1	2	3	4	5

7. If you had to spend the rest of your life with your **heart failure** the way it is right now, how would you feel about this?

Not at all satisfied	Mostly dissatisfied	Somewhat satisfied	Mostly satisfied	Completely satisfied
O	O	O	O	O
1	2	3	4	5

8. How much does your **heart failure** affect your lifestyle? Please indicate how your **heart failure** may have limited your participation in the following activities over the past 2 weeks.

**Activity**	**Severely** Limited	Limited **quite a bit**	**Moderately** limited	**Slightly** limited	**Did not** limit at all	Does not apply or did not do for other reasons
a. Hobbies, recreational activities	O	O	O	O	O	O
b. Working or doing household chores	O	O	O	O	O	O
c. Visiting family or friends out of your home	O	O	O	O	O	O
	1	2	3	4	5	6

© 2026 Eberly L et al. *JAMA Network Open*.

Nutrition security:

14. Food security-6 item USDA

Here are some statements that people have made about their food situation. For these statements, please tell me whether the statement was often true, sometimes true, or never true for (you/your household) in the last 12 months—that is, since last (name of current month).

In the last 12 months, the food that we bought just didn't last, and we didn't have money to get more.	☐ Often true<br>☐ Sometimes true<br>☐ Never true<br>☐ Don't know or prefer not to answer
In the last 12 months, we couldn't afford to eat balanced meals	☐ Often true<br>☐ Sometimes true<br>☐ Never true<br>☐ Don't know
In the last 12 months, did you ever eat less than you felt you should because there wasn't enough money for food?	☐ Yes<br>☐ No<br>☐ Don't know
In the last 12 months, were you ever hungry but didn't eat because there wasn't enough money for food?	☐ Yes<br>☐ No<br>☐ Don't know
In the last 12 months, since last (name of current month), did you or other adults in your household ever cut the size of your meals or skip meals because there wasn't enough money for food?	☐ Yes<br>☐ No → Skip to Question 15<br>☐ Don't know → Skip to Question 15
If you answered yes to the last question, how often did this happen—almost every month, some months but not every month, or in only 1 or 2 months?	☐ Almost every month<br>☐ Some months but not every month<br>☐ Only 1 or 2 months<br>☐ Don't know

© 2026 Eberly L et al. *JAMA Network Open*.

15. Which of the following programs have you or someone in your household used in the last 30-days?

☐1 Food stamps (SNAP) ☐2 Commodities (FDPIR) ☐3 WIC
☐4 Cash Assistance/TANF ☐5 DES Child Care Subsidy ☐6 Quality First Scholarship
☐7 Other (Please specify: ___________) ☐8 None of the above

16. Diet Quality (DSQ-10):

(10-item DSQ with additional Navajo specific question)
16.1 During the past month, how often did you drink 100% PURE FRUIT JUICES such as orange, apple, grape, etc.? DO NOT INCLUDE fruit-flavored drinks with added sugars like Capri-Sun, Sunny D, or other fruit-flavored drinks?

1. Never
2. 1 time last month
3. 2 – 3 times last month
4. 1 time per week
5. 2 times per week
6. 3 – 4 times per week
7. 5 – 6 times per week
8. 1 time per day
9. 2 – 3 times per day
10. 4 – 5 times per day
11. 6 or more times per day
12. Don't know/prefer not to answer

16.2 During the past month, how often did you eat FRUITS like apples, bananas, oranges, melon, or any other fruits? INCLUDE fresh, frozen, canned, or dried fruit. DO NOT INCLUDE juices

- Never
- 1 time last month
- 2-3 times last month
- 1 time per week
- 2 times per week
- 3-4 times per week
- 5-6 times per week
- 1 time per day
- 2 or more times per day
- Don't know/Prefer not to answer

16.3 During the past month, how often did you eat a GREEN LEAFY OR LETTUCE SALAD, with or without other vegetables?

- Never
- 1 time last month
- 2-3 times last month
- 1 time per week
- 2 times per week
- 3-4 times per week
- 5-6 times per week
- 1 time per day
- 2 or more times per day
- Don't know/Prefer not to answer

16.4 During the past month, how often did you eat any kind of FRIED POTATOES like French fries, tater tots, hash brown potatoes, or other fried potatoes?

- Never
- 1 time last month
- 2-3 times last month
- 1 time per week
- 2 times per week
- 3-4 times per week
- 5-6 times per week

© 2026 Eberly L et al. *JAMA Network Open*.

- 1 time per day
- 2 or more times per day
- Don’t know/Prefer not to answer

16.5 During the past month, how often did you eat ANY OTHER KIND OF POTATOES that aren’t fried like baked, boiled, mashed, or potatoes used in soups or stews

- Never
- 1 time last month
- 2-3 times last month
- 1 time per week
- 2 times per week
- 3-4 times per week
- 5-6 times per week
- 1 time per day
- 2 or more times per day
- Don’t know/Prefer not to answer

16.6 During the past month, how often did you eat refried beans, baked beans, pinto beans, black beans, beans in soup, or any other type of COOKED BEANS? INCLUDE canned or dry beans. DO NOT INCLUDE green beans or string beans

- Never
- 1 time last month
- 2-3 times last month
- 1 time per week
- 2 times per week
- 3-4 times per week
- 5-6 times per week
- 1 time per day
- 2 or more times per day
- Don’t know/Prefer not to answer

16.7 During the past month, how often did you eat other VEGETABLES that were not deepfried? These are vegetables like carrots, broccoli, collards, green beans, corn, or other vegetables that are not deep-fried. INCLUDE canned, frozen, and fresh vegetables. ALSO INCLUDE vegetables that are raw, boiled, broiled, baked, grilled, stir-fried, or microwaved.

- Never
- 1 time last month
- 2-3 times last month
- 1 time per week
- 2 times per week
- 3-4 times per week
- 5-6 times per week
- 1 time per day
- 2 or more times per day
- Don’t know/Prefer not to answer

16.8 During the past month, how often did you eat packaged or homemade SALSA made with tomato?

- Never
- 1 time last month
- 2-3 times last month
- 1 time per week
- 2 times per week
- 3-4 times per week
- 5-6 times per week
- 1 time per day
- 2 or more times per day
- Don’t know/Prefer not to answer

16.9 During the past month, how often did you eat PIZZA? INCLUDE frozen pizza, fast food pizza, and homemade pizza

- Never
- 1 time last month
- 2-3 times last month
- 1 time per week

© 2026 Eberly L et al. *JAMA Network Open*.

- 2 times per week
- 3-4 times per week
- 5-6 times per week
- 1 time per day
- 2 or more times per day
- Don’t know/Prefer not to answer

16.10 During the past month, how often did you eat TOMATO SAUCE in recipes such as spaghetti, lasagna, or other dishes? DO NOT INCLUDE tomato sauce on pizza

1. Never

- 1 time last month
- 2-3 times last month
- 1 time per week
- 2 times per week
- 3-4 times per week
- 5-6 times per week
- 1 time per day
- 2 or more times per day
- Don’t know/Prefer not to answer

16.11 During the past month, how often did you eat traditional Diné foods (such as blue corn mush, steamed, roasted or dried corn, sumac berries, mutton, local varieties of squash or beans):

1. Never
2. 1 time last month
3. 2 – 3 times last month
4. 1 time per week
5. 2 times per week
6. 3 – 4 times per week
7. 5 – 6 times per week
8. 1 time per day
9. 2 – 3 times per day
10. 4 – 5 times per day
11. 6 or more times per day

<u>Food preferences/restrictions:</u>

17. How many meals a day do you typically eat?

    [ ] One [ ] Two [ ] Three [ ] Other______________________________

18. If food availability or cost of food was not a concern, how many meals a day would you eat a day?

    [ ] One [ ] Two [ ] Three [ ] Other______________________________

19. Any food allergies? ______________________

20. Please check ALL of the foods that you are ok eating:

☐ Dairy	☐ Fish / Seafood	☐ Mutton / Lamb	☐ Chicken / Turkey	☐ Pork	☐ Beef
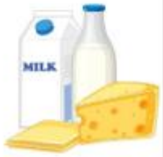	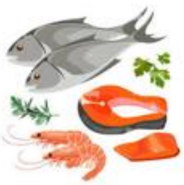	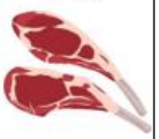	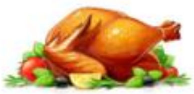	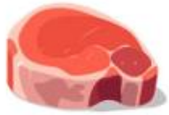	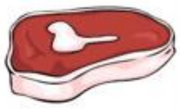

© 2026 Eberly L et al. *JAMA Network Open*.

21. Do you prefer Traditional Navajo foods or non-traditional meals?

    a. [ ] Traditional. [ ] Nontraditional. [ ] Mix of both

<u>Food delivery preferences</u>

22. Are you (or a family member) able to pick up meals every week at the Gallup Food Pantry?

    (Gallup Pantry is located in town near Playground of Dreams/The Bus Station, right off exit 22 off I-40)

    [ ] Yes → Please list the names of anyone you designate to pick up meals on your behalf:

    ____________________ ____________________

    → Skip to question 25

    [ ] No → continue to next question

23. (If you are unable to pick up at Gallup Pantry), are you (or a family member) able to pick up meals at your local Chapter House/Senior Center?

    [ ] Yes → Please list the names of anyone you designate to pick up meals on your behalf:

    ____________________ ____________________

    ____________________ ____________________

    → Skip to question 25

    [ ] No → continue to next question

24. (If you are unable to pick up at a Chapter House / Senior Center), are you able to receive meal deliveries to your home every week?

    [ ] Yes → Since someone needs to be home for delivery, list days / times of day meals can be delivered:

    ____

    [ ] No → Is there a location that we could deliver meals to you (i.e. work, church, family member's home)?

    ____

25. In the event you (or a family member) are unable to pick up the meals, do you designate a CHR to pick up the meals and delivery to you if you (or your family members) are unable to pick up?

    [ ] Yes [ ] No

© 2026 Eberly L et al. *JAMA Network Open*.

Resource assessment

26. How often does this describe you? I don't have money to pay my bills (including food, housing, medical care or heating). Never / Rarely / Sometimes / Often / Always

27. We are interested in knowing what resources you have in your home. Please check all of the following that you have:

☐ Working microwave 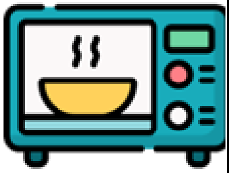	☐ Working stove 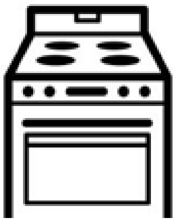	☐ Working freezer 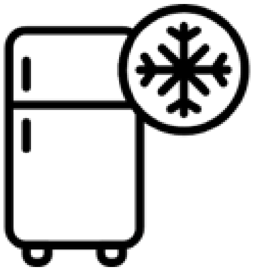	☐ Working refrigerator 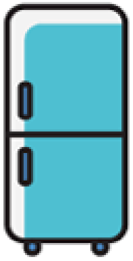	Reliable Electricity 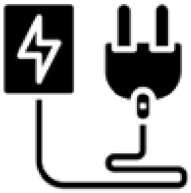	☐ Running (tap) water 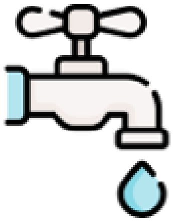

If you have running (tap) water in your home, please answer the following questions:

"My tap water at home is safe to drink"	☐ Disagree<br>☐ Neither agree nor disagree<br>☐ Agree<br>☐ Not sure / prefer not to answer
"My tap water at home is safe to cook with,"	☐ Disagree<br>☐ Neither agree nor disagree<br>☐ Agree<br>☐ Not sure / prefer not to answer
When you drink tap water, what is the main source of the tap water?	☐ City or NTUA water supply<br>☐ Well or rain cistern<br>☐ Spring<br>☐ Other source:________________<br>☐ I never drink tap water

© 2026 Eberly L et al. *JAMA Network Open*.

28. If you have a freezer, which looks most like the freezer at your home? (mark all that apply if have more than 1)

☐ Family size with top, bottom or side freezer (~3-6 cubic feet) 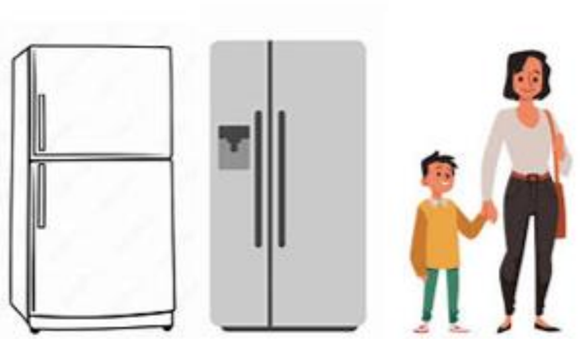	☐ Mini fridge with freezer (~1 cubic feet freezer space) 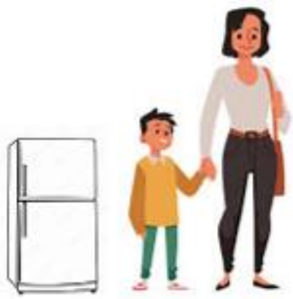	☐ Freezer chest 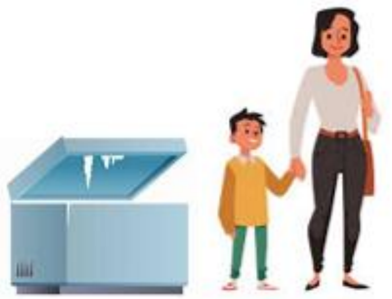
In your freezer(s), how much freezer space is **available (empty) space** for storing meals?		☐ **None** of the freezer space<br>☐ **About half** the freezer space<br>☐ **Most** of the freezer space<br>☐ **All** of the freezer space

29. Finally, we would like to know what community resources you engage with. Check all services or community resources that you use or engage with regularly:

    [ ] Senior center

    [ ] Meals on wheels

    [ ] Public Health Nurse

    [ ] Community Health Representative

    [ ] Local church

    [ ] Other ____

© 2026 Eberly L et al. *JAMA Network Open*.

eAppendix 2. Patient Postintervention Survey

Participant ID/Initials ________________________ Date ____

Who filled out this form_________________________________

How was form filled out: [ ] over the phone [ ] in person

1. CCS-S

How strongly do you agree with these statements?	Strongly agree	Agree	Don't agree or disagree	Disagree	Strongly disagree
In certain situations, I believe things like animals and rocks have a spirit like Diné people	☐	☐	☐	☐	☐
I plan to learn more about my Diné culture, such as its history, traditions and customs	☐	☐	☐	☐	☐
I have a traditional person or Elder who I talk to about Diné beliefs and culture	☐	☐	☐	☐	☐
I have spent time trying to find out more about being Navajo, such as my history, traditions and customs	☐	☐	☐	☐	☐
I feel like I belong to the Diné community	☐	☐	☐	☐	☐
I feel a strong attachment towards my Diné community	☐	☐	☐	☐	☐
The eagle feather has a lot of meaning to me	☐	☐	☐	☐	☐
I use mountain tobacco for guidance	☐	☐	☐	☐	☐
How many of your clans can you name?	☐ 1	☐ 2	☐ 3	☐ 4	☐ Not sure
How often does someone in your family or someone you are close with use sage, cedar or other Diné traditional herbs?	☐ Every day	☐ Every week	☐ Every month	☐ 1-2 times a year	☐ Never

Health questions

30. Would you say that in general your health is excellent, very good, good, fair, or poor?

[ ] Excellent [ ] Very Good [ ] Good [ ] Fair [ ] Poor

© 2026 Eberly L et al. *JAMA Network Open*.

31. How many minutes would you estimate you exercise weekly?________________

32. Kansas City Cardiomyopathy Questionnaire (KCCQ-12)

Kansas City Cardiomyopathy Questionnaire (KCCQ-12)

The following questions refer to your **heart failure** and how it may affect your life. Please read and complete the following questions. There are no right or wrong answers. Please mark the answer that best applies to you.

1. **Heart failure** affects different people in different ways. Some feel shortness of breath while others feel fatigue. Please indicate how much you are limited by **heart failure** (shortness of breath or fatigue) in your ability to do the following activities over the past 2 weeks.

**Activity**	**Extremely** Limited	**Quite a bit** Limited	**Moderately** Limited	**Slightly** Limited	**Not at all** Limited	Limited for other reasons or did not do the activity
a. Showering/bathing	O	O	O	O	O	O
b. Walking 1 block on level ground	O	O	O	O	O	O
c. Hurrying or jogging (as if to catch a bus)	O	O	O	O	O	O
	1	2	3	4	5	6

2. Over the past 2 weeks, how many times did you have **swelling** in your feet, ankles or legs when you woke up in the morning?

Every morning	3 or more times per week but not every day	1-2 times per week	Less than once a week	Never over the past 2 weeks
O	O	O	O	O
1	2	3	4	5

3. Over the past 2 weeks, on average, how many times has **fatigue** limited your ability to do what you wanted?

All of the time	Several times per day	At least once a day	3 or more times per week but not every day	1-2 times per week	Less than once a week	Never over the past 2 weeks
O	O	O	O	O	O	O
1	2	3	4	5	6	7

4. Over the past 2 weeks, on average, how many times has **shortness of breath** limited your ability to do what you wanted?

All of the time	Several times per day	At least once a day	3 or more times per week but not every day	1-2 times per week	Less than once a week	Never over the past 2 weeks
O	O	O	O	O	O	O
1	2	3	4	5	6	7

5. Over the past 2 weeks, on average, how many times have you been forced to sleep sitting up in a chair or with at least 3 pillows to prop you up because of **shortness of breath**?

Every night	3 or more times per week but not every day	1-2 times per week	Less than once a week	Never over the past 2 weeks
O	O	O	O	O
1	2	3	4	5

[illegible]

© 2026 Eberly L et al. *JAMA Network Open*.

6. Over the <u>past 2 weeks</u>, how much has your **heart failure** limited your enjoyment of life?

It has **extremely** limited my enjoyment of life	It has limited my enjoyment of life **quite a bit**	It has **moderately** limited my enjoyment of life	It has **slightly** limited my enjoyment of life	It has **not limited** my enjoyment of life at all
O	O	O	O	O
1	2	3	4	5

7. If you had to spend the rest of your life with your **heart failure** the way it is <u>right now</u>, how would you feel about this?

Not at all satisfied	Mostly dissatisfied	Somewhat satisfied	Mostly satisfied	Completely satisfied
O	O	O	O	O
1	2	3	4	5

8. How much does your **heart failure** affect your lifestyle? Please indicate how your **heart failure** may have limited your participation in the following activities <u>over the past 2 weeks</u>.

**Activity**	**Severely** Limited	Limited **quite a bit**	**Moderately** limited	**Slightly** limited	**Did not** limit at all	Does not apply or did not do for other reasons
a. Hobbies, recreational activities	O	O	O	O	O	O
b. Working or doing household chores	O	O	O	O	O	O
c. Visiting family or friends out of your home	O	O	O	O	O	O
	1	2	3	4	5	6

<u>Nutrition security:</u>

33. Food security-6 item USDA

Here are some statements that people have made about their food situation. For these statements, please tell me whether the statement was often true, sometimes true, or never true for (you/your household) in the last 1 month ONLY.

In the last 1 month, the food that we bought just didn't last, and we didn't have money to get more.	☐ Often true<br>☐ Sometimes true<br>☐ Never true<br>☐ Don't know or prefer not to answer
In the last 1 month, we couldn't afford to eat balanced meals	☐ Often true<br>☐ Sometimes true<br>☐ Never true<br>☐ Don't know

© 2026 Eberly L et al. *JAMA Network Open*.

In the last 1 month, did you ever eat less than you felt you should because there wasn't enough money for food?	☐ Yes<br>☐ No<br>☐ Don't know
In the last 1 month, were you every hungry but didn't eat because there wasn't enough money for food?	☐ Yes<br>☐ No<br>☐ Don't know
In the last 1 month, did you or other adults in your household ever cut the size of your meals or skip meals because there wasn't enough money for food?	☐ Yes<br>☐ No → Skip to Question 5<br>☐ Don't know → Skip to Question 5
If you answered yes to the last question, how often did this happen—almost every week, some weeks but not every week, or in only 1 or 2 times?	☐ Almost every week<br>☐ Some weeks but not every week<br>☐ Only 1 or 2 times<br>☐ Don't know

34. We'd love your honest feedback on the meal program. This information will help us improve the program in the future.

1. How would you rate the meals overall, in terms of taste?

   [ ] Excellent
   [ ] Good
   [ ] Average
   [ ] Below average
   [ ] Bad

2. In terms of sharing meals, how much of the meals were shared with others?

   [ ] All
   [ ] Most
   [ ] About half
   [ ] Less than half
   [ ] None

3. In terms of leftovers, how much of the meals were not eaten and had to be thrown out?

   [ ] All
   [ ] Most
   [ ] About half
   [ ] Less than half
   [ ] None

4. I would rate getting the meals (either picking up or delivery)

   [ ] Very easy
   [ ] Somewhat easy
   [ ] Neither easy nor difficult
   [ ] Somewhat difficult
   [ ] Very difficult

© 2026 Eberly L et al. *JAMA Network Open*.

35. How likely is it that this program will change your diet to be healthier moving forward?

[ ] Very likely
[ ] Somewhat likely
[ ] Neither likely nor unlikely
[ ] Somewhat unlikely
[ ] Very unlikely

36. How likely is it that you will add any of the meals or recipes from this program to your on-going diet in the future?

[ ] Very likely
[ ] Somewhat likely
[ ] Neither likely nor unlikely
[ ] Somewhat unlikely
[ ] Very unlikely

37. Acceptability of the Program Measure

Please tell us how much you agree with the following statements:

	Completely disagree	Disagree	Neither agree nor disagree	Agree	Completely Agree
6.1 I approve of this food is medicine program					
6.2 The food is medicine program is appealing to me					
6.3 I like the food is medicine program					
6.4 I welcome having more of this food is medicine program					

38. From 1-10 (with 1 being highly unlikely and 10 highly likely), how likely is it that you would recommend this program to a community member?

____

eAppendix 3. Semistructured Interview Questions for Patients

A. Tell me about your experience being a part of the food program?

a. Can be prompted further if needed: “What did you like about the program? What did you not like about the food program?”

B. Tell me about your experience getting the meals (i.e. picking up or having meals delivered to you?)

C. If you could change one or two things in regard to the food program, what would you change?

D. Anything else to share?

© 2026 Eberly L et al. *JAMA Network Open*.

eAppendix 4. Semistructured Interview Questions for Farmers and Ranchers

Thank you for agreeing to participate. We are hoping to highlight local farmers and ranchers, as well as learn more about how it went participating in our food program so we can think about how to best support you as we plan next steps to expand the program.

Question 1: What do you grow or produce?

Question 2: How did you learn to grow or produce this food?

Question 3: What is your favorite part of growing or producing this food?

Question 4: Are there any words of encouragement you'd like to add for our community or youth to hear?

The next few questions will be about your experience in the food is medicine program.

Please let us know how much you agree with the following statements. (Sharon-check off response):

	Completely disagree	Disagree	Neither agree nor disagree	Agree	Completely Agree
6.1 Supplying produce (or meat) for the food program seems manageable					
6.2 Supplying produce (or meat) for the food program seems possible					
6.3 Supplying produce (or meat) for the food program seems doable					
6.4 Supplying produce (or meat) for the food program seems easy					

1. Tell me about your experience being a part of the food program?
    1. "What did you like about the program? What did you not like about the food program?"
2. Tell me about your experience getting your produce (or meat) to the program?
3. If you could change one or two things in regard to the food program, what would you change?
4. Could you share some of the challenges you might face as a farmer/rancher supplying this program?
    1. And what might make those easier?
5. Anything else to share?

© 2026 Eberly L et al. *JAMA Network Open*.
