# Supplementary material for: Feasibility of an Indigenous Food Is Medicine Program for Patients With Heart Failure in Rural Navajo Nation: The MUTTON-HF Nonrandomized Clinical Trial
Source: JAMA Netw Open. 2026 Feb 6;9(2):e2556117. doi: 10.1001/jamanetworkopen.2025.56117 (PMC12881988; doi:10.1001/jamanetworkopen.2025.56117)
Supplement: Supplement 3. — Data Sharing Statement [file jamanetwopen-e2556117-s003.pdf]

# Data Sharing Statement

Eberly. Feasibility of an Indigenous Food Is Medicine Program for Patients With Heart Failure in Rural Navajo Nation. *JAMA Netw Open*. Published February 06, 2026. doi:10.1001/jamanetworkopen.2025.56117

**Data**
**Additional Information:** https://clinicaltrials.gov/study/NCT06675331 NCT06675331
**Data available:** No

**Additional Information**
**Explanation for why data not available:** Per Navajo Nation Human Research Review Board and tribal regulations, data is owned by the Navajo tribe and can be requested from the Navajo Nation Human Research Review Board.
